# Experimental *Sodalis* infection eliminates ancient insect symbiont

**DOI:** 10.1038/s41467-026-71143-2

**Published:** 2026-03-31

**Authors:** Ronja Krüsemer, Ana S. P. Carvalho, Jean Keller, Heiko Vogel, Colin Dale, Tobias Engl, Martin Kaltenpoth

**Affiliations:** 1 Department of Insect Symbiosis, Max Planck Institute for Chemical Ecology, Jena, Germany; 2 Laboratoire de Recherche en Sciences Végétales, Université de Toulouse, CNRS, UPS, Toulouse INP, Castanet-Tolosan, France; 3 School of Biological Sciences, University of Utah, Salt Lake City, UT USA

**Keywords:** Coevolution, Symbiosis, Microbial ecology, Evolutionary ecology, Entomology

## Abstract

Many insects benefit from ancient nutrient-supplementing endosymbionts. While symbiont losses and replacements occur on evolutionary timescales, their dynamics remain enigmatic due to the lack of experimentally tractable systems. Here, we report on the experimental establishment of the culturable bacterium *Sodalis praecaptivus* in a grain pest beetle (*Oryzaephilus surinamensis*) and its effect on the native symbiont *Shikimatogenerans silvanidophilus*, which produces the tyrosine precursor prephenate. Injection of *Sodalis* into female beetles led to systemic intracellular infection and efficient transovarial vertical transmission but reduced host survival and reproduction. Interestingly, *Sodalis* also invaded the host’s bacteriomes, causing irregular morphology and rapid loss of *Shikimatogenerans* within three beetle generations. Transcriptomics revealed a strong upregulation of host immune effectors upon *Sodalis* infection, but little reaction from *Shikimatogenerans*, indicating that the ancient symbiont is incapable of responding adaptively to the introduced competitor. The rapid elimination of the native symbiont in *O. surinamensis* showcases the fragility of ancient beneficial symbioses and experimentally recapitulates a crucial step towards a functional symbiont replacement.

## Abstract (web summary)

Many insects harbour bacterial endosymbionts that provide them with essential nutrients. Here, Krüsemer et al. show that introduction of a tractable bacterium into a beetle host leads to efficient vertical transmission of the bacterium and rapid elimination of the host’s native symbiont, thus establishing a model system to study symbiont loss and replacement.

## Introduction

Insects commonly engage in intracellular symbioses with microorganisms spanning the continuum from pathogens to mutualists. Associations with beneficial symbionts in particular play an important role in insect adaptation and diversification, because the biosynthetic capabilities of microbial symbionts can provide vital metabolites to the host, such as essential nutrients, digestive or detoxifying enzymes, or protective secondary metabolites, thereby enabling insects to expand into otherwise inaccessible ecological niches^1,2^. Many interactions between insects and beneficial microorganisms are ancient in origin^3–6^ and have experienced tight coevolution. This process commonly results in the erosion of the symbiont genome^7^ and ultimately leads to mutual metabolic dependence of the symbiotic partners^8^. Despite the intimacy and functional importance of many intracellular insect symbioses, phylogenetic analyses reveal that ancient beneficial symbionts are occasionally lost^9,10^ or replaced by other microorganisms^11–14^.

Symbiont losses can occur when symbiont-provided benefits no longer outweigh the costs of maintaining them. In insects depending on symbiotic microbes to compensate for nutritional deficiencies^2^, the loss of a costly symbiont can be advantageous for hosts that acquire the metabolic capacity to perform symbiont-mediated reactions themselves, for example via horizontal gene transfer^10^. Alternatively, symbiont losses may be catalyzed by changes in the host’s ecological niche—e.g., a switch to a more nutritionally balanced diet—that render symbiont-provided benefits unnecessary^15^. Finally, a newly acquired symbiont can lead to the demise of the original one by replacing or even expanding the repertoire of benefits provided to the host. Such symbiont replacements are often assumed to be beneficial to the host, as they allow for escaping the rabbit hole of increasing genome erosion and accumulation of slightly deleterious mutations in the symbiont’s genome due to Muller’s ratchet^12^.

For a replacement of an intracellular symbiont to be successful, four criteria have to be fulfilled: The new symbiont must be able to (i) colonize and persist in the host, and (ii) achieve efficient transmission. Additionally, (iii) the original symbiont must be eliminated, while (iv) the newly acquired symbiont evolves a net benefit and takes over (and potentially extends) the original symbiont’s role. In contrast to unicellular systems, like ciliates, where symbiont replacements have been well studied^16^ and can be experimentally recapitulated^17,18^, the sequence of events leading to a replacement of an intracellular symbiont and the speed at which they occur remain obscure in animals, where the soma-germline separation constitutes a considerable barrier for vertical transmission. In insects, symbiont replacements are widespread but rare on ecological timescales, and most intracellular symbioses are intractable, hampering experimental studies that address symbiont losses and replacements despite their importance in understanding the evolutionary dynamics of host-microbe interactions^19^.

Intracellular associations that may enable experimental symbiont replacements have recently been described in several beetle lineages. Coleoptera, the most species-rich order of insects^20^, are characterized by the rigid elytra that protect the beetle from desiccation and predation^21^. To build this protective barrier, however, beetles require large amounts of tyrosine, because tyrosine derivatives like *N*-acetyldopamine, *N*-β-alanyldopamine, and melanin are necessary for cuticle sclerotization and melanization^22,23^. Tyrosine is a semi-essential amino acid that insects cannot synthesize de novo and therefore need to acquire from the diet or from symbiotic microorganisms. Concordantly, to meet their high demands for tyrosine, many beetles associate with intracellular symbionts that supply tyrosine precursors and thereby support cuticle development under nutrient-limited conditions^6,9^. These tyrosine-supplementing symbioses are promising candidates for establishing experimentally tractable symbiotic systems, since many tyrosine-producing symbionts are not essential for host survival under laboratory conditions^24–26^. This can allow for the functional replacement of a beneficial symbiont with a culturable bacterium, as demonstrated by the experimental replacement of *Sitophilus zeamais’* native symbiont *Sodalis pierantonius* with the culturable congener *Sodalis praecaptivus*^27^ that was engineered to overproduce and secrete tyrosine^19^.

Here, we explored the potential of *S. praecaptivus* (from here on referred to as *Sodalis*) to establish successful infections in the saw-toothed grain beetle *Oryzaephilus surinamensis* harboring the distantly related *Bacteroidota* symbiont *Shikimatogenerans silvanidophilus* (from here on referred to as *Shikimatogenerans*). *Shikimatogenerans* bacteria are harbored intracellularly in two pairs of bacteriomes and are transmitted vertically from mother to offspring. The symbiotic bacteria provision the tyrosine precursor prephenate to their host, which the beetle converts into tyrosine to support cuticle biosynthesis^6,28,29^. We demonstrate that, upon injection into adult females, *Sodalis* establishes systemic intracellular infections in the novel host. Importantly, the new symbiont spontaneously achieves highly efficient vertical transmission to the offspring, a feature that is widely accepted to be crucial for the evolution of beneficial symbioses by aligning host and symbiont fitness interests^30,31^. However, in the initial encounter between *Oryzaephilus* and *Sodalis*, the latter negatively impacts host fitness. We further show that the presence of *Sodalis* disrupts the native symbiont, affects its morphology, and ultimately leads to its rapid loss from the host population within three beetle generations. Triple RNAseq reveals that the host mounts a strong immune response to *Sodalis’* presence in the bacteriome tissue, but *Shikimatogenerans* is incapable of adjusting its gene expression in response to the intruder. Our results show that infection with a novel facultative symbiont can result in the rapid breakdown of an ancient beneficial symbiosis and lead to the elimination of the native symbiont, thus fulfilling three out of the four criteria necessary for a stable symbiont replacement. Despite the detrimental nature of the novel symbiont, its maintenance and highly efficient vertical transmission may pave the way for a symbiont replacement in the future, thus providing an experimentally tractable system to recapitulate major events in the evolution of beneficial symbioses in insects.

## Results

### *Sodalis* establishes systemic infection in *O. surinamensis* and is vertically transmitted

**Fig. 1:**
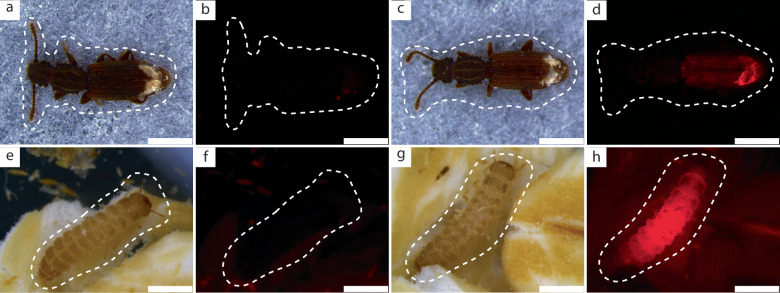
*Sodalis* establishes in injected beetles and and is transmitted to their offspring. Brightfield images of injected control (**a**) and *Sodalis*-treated females (**c**) and their corresponding fluorescence microscopy images (**b**, **d**) taken 7 days after injection. Note that the distal ends of the elytra were removed for injection, facilitating detection of fluorescence at the posterior end of the abdomen. Brightfield images of first-generation larval offspring of control (**e**) and *Sodalis*-injected (**g**) females and their corresponding fluorescence microscopy images (**f**, **h**). Red signal in **d**, **h** is indicative of mCherry protein produced by *Sodalis*. Fluorescence deriving from *Sodalis* infection was detected in all females injected with *Sodalis* that survived up to 7 dpi (*n* = 297) while it was absent from all control beetles surviving up to this timepoint (*n* = 305). No fluorescence was observed in larvae of the control treatment (*n* = 1555), but could be detected in a majority of larvae produced by *Sodalis*-infected females (*n* = 227). Scale bars = 1 mm.

We tested the potential of fluorescently labeled *Sodalis* to establish a novel symbiotic interaction with *O. surinamensis* beetles by microinjecting 10 nl aliquots of a *Sodalis* suspension (OD_600_ = 1, i.e., 6.01 × 10^3^ ± 2.79 × 10^3^ CFUs) into the abdomen of reproductively active female adults. After 7 days, we observed that beetles injected with *Sodalis* exhibited strong fluorescence, indicative of mCherry production by this bacterium (Fig. 1a–d). Uninfected control beetles were devoid of mCherry fluorescence, verifying that the signal observed was not caused by cuticular autofluorescence. As determined via quantitative polymerase chain reaction (qPCR), the population of *Sodalis* had grown to 1.46 × 10^7^ ± 7.28 × 10^6^ *tam *gene copy numbers by day 7 and slightly increased even further in the following week (2.38 × 10^7^ ± 9.12 × 10^6^ *tam* gene copy numbers on day 14, Supplementary Fig. 1).

When screening larval first generation (F1) offspring, we discovered that offspring from females injected with bacteria also showed strong mCherry fluorescence, demonstrating that females harboring *Sodalis* vertically transmitted the novel symbionts to their offspring (Fig. 1e–h). With fluorescence-in-situ-hybridization (FISH), we confirmed that transmission indeed occurred vertically via the ovaries, since we detected *Sodalis* cells in young oocytes (Supplementary Fig. 2). Transmission occurred with an efficiency of 93.75% (Supplementary Fig. 3). We did not observe any paternal transmission of *Sodalis*: Larvae produced by mating pairs of *Sodalis-*infected virgin males and uninfected virgin females never exhibited mCherry fluorescence (Supplementary Fig. 4). Males harboring *Sodalis* also did not transmit the bacteria to the female beetles during mating (Supplementary Fig. 4). We were able to maintain *Sodalis* infected beetles up to the third offspring generation by mating infected males and females, before sacrificing all remaining specimens for microscopy.

### *Sodalis* infection negatively affects host fitness

**Fig. 2:**
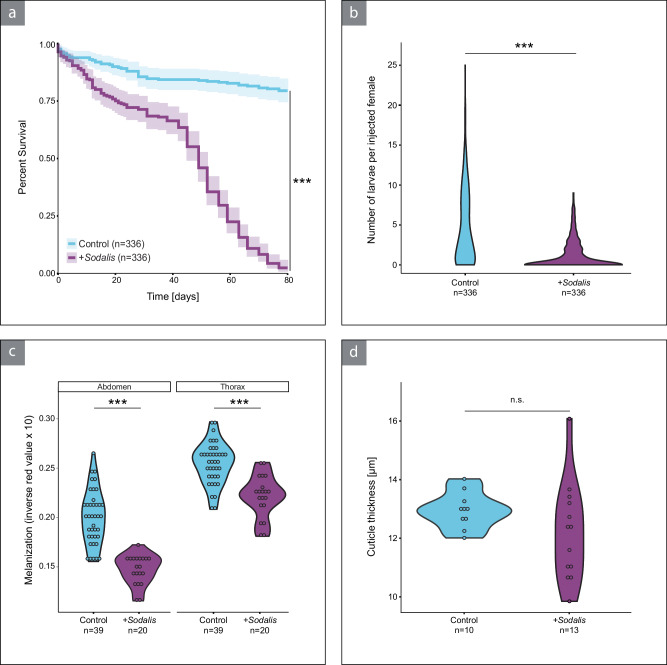
Infection with *Sodalis* has a negative impact on host fitness. **a** Survival probability (mean and 95% confidence interval) of control (light blue) and *Sodalis* injected beetles (purple). The presence of *Sodalis* negatively affected beetle survival probability over the observation period (Cox mixed-effects model, ANOVA, *P* < 2.2 × 10^−16^). **b** The number of larvae produced by each female injected with *Sodalis* (purple) was significantly lower than in control females (light blue, zero-inflated negative binomial model, Wald *z*-test, *P* < 2.2 × 10^−16^). **c** 7-day old adults of first-generation offspring had significantly less melanized cuticles of both thorax and abdomen when they harbored *Sodalis* (thorax: two-sided two sample *t*-test, *P* = 3.49 × 10^−7^, abdomen: two-sided Wilcoxon rank sum test, *P* = 1.23 × 10^−10^). **d** Cuticle thickness in thorax sections did not differ in 7-day old adults of first-generation offspring between both treatments (two-sided Wilcoxon rank sum-test, *P* = 0.13). Statistical differences indicated by asterisks (^***^P < 0.001). n.s.: not significant. Source Data are provided as a Source Data file.

Following the successful establishment of *Sodalis* in *O. surinamensis* beetles, we investigated the impact on host fitness. While 80% of control beetles survived the observation period of 80 days, *Sodalis* infection significantly reduced beetle survival (Cox mixed-effects model, df = 1, *Χ*^2^ = 214.31, *P* < 0.001, Fig. 2a). Almost no beetles harboring the novel symbiont survived the observation period, and over 50% of individuals had died by day 50. We also found beetles carrying *Sodalis* to produce significantly fewer larvae than beetles of the control treatment (zero-inflated negative binomial model, *z* = −9.46, *P* < 0.001, Fig. 2b). For the F1 adults, we investigated cuticle melanization and thickness as indicators for tyrosine availability and proxies for host fitness with respect to protection from predation and desiccation. Beetles harboring *Sodalis* were significantly lighter than their control counterparts in both abdomen (Wilcoxon rank sum test, *W* = 741, *P* < 0.001) and thorax (two sample *t*-test, df = 57, *t* = 5.76, *P* < 0.001), suggesting a reduced amount of tyrosine available for melanization (Fig. 2c). In contrast to melanization, cuticle thickness was not affected by *Sodalis* infection, independent of whether we measured in individual sections (Wilcoxon rank sum test, *W* = 90, *P* = 0.13, Fig. 2d) or whole beetles (two-sample *t*-test, df = 20. *t* = −0.85, *P* = 0.41, Supplementary Fig. 5).

### *Sodalis-*infected beetles lose their native symbiont within three generations

**Fig. 3:**
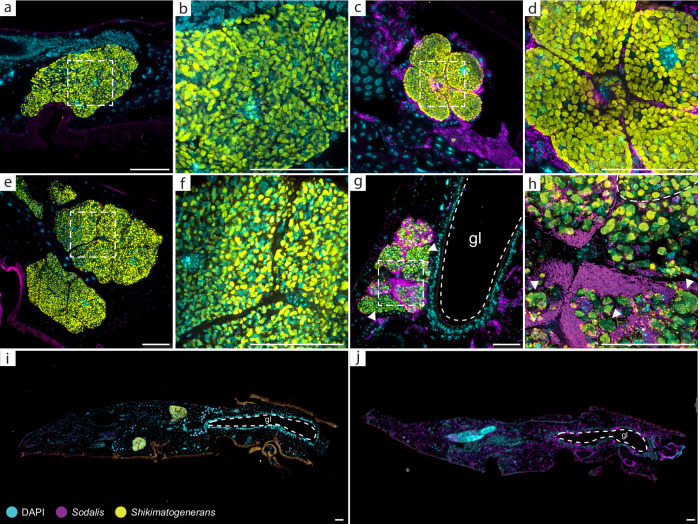
*Sodalis* invades the host bacteriomes where it causes aberrant *Shikimatogenerans* morphology, and, ultimately, loss of the native symbiont. FISH micrographs of control (**a**, **b**) and *Sodalis* injected beetles (**c**, **d**) of the parental generation, **b** and **d** are closeups of the areas marked with a frame in **a** and **c**. In 7-day old beetles of the first offspring generation, *Shikimatogenerans* displayed globular or rod-shaped morphology in *Sodalis*-free control beetles (**e**, **f**), while symbiont cells were aberrantly shaped upon colocalization with *Sodalis* (**g**, **h**). **f** and **h** are closeups of the areas marked with a frame in **e** and **g**. Arrows in **g** highlight bacteriocytes that are almost uninfected with *Sodalis*. Arrows in **h** highlight particularly altered *Shikimatogenerans* morphology, while the dashed area highlights regularly shaped symbiont cells in a practically uninfected bacteriocyte of the same bacteriome. In adults of the third offspring generation, *Shikimatogenerans* was present in the control treatment (**i**), while it was lost in adults harboring *Sodalis* (**j**). DNA was stained with DAPI (cyan), while the probes OsurSym16S-Cy5 and Sod-FISH-Cy3 were used to label *Shikimatogenerans* (yellow) and *Sodalis* (magenta), respectively. Observations were replicated independently with similar results (Parental generation: *n* = 5 for either treatment, F1: *n* = 7 for control and *n* = 10 for *Sodalis*-infected beetles, F3(+): *n* = 10 for control and *n* = 6 for *Sodalis*-infected beetles). Scale bars represent 50 µm, gl= gut lumen.

We performed FISH to investigate the localization of *Sodalis* in *O. surinamensis* across three different host generations. While *Sodalis* was consistently absent from control beetles (Fig. 3a, b, e, f, i, and Supplementary Figs. 6–8), it occupied various tissues in injected beetles and their offspring. In the parental beetle generation, the novel symbiont was found in the hemolymph and fatbody across the whole host body, the ventral nerve cord, and was also highly abundant in the ovaries. We also found *Sodalis* in the bacteriomes, even though it was less abundant than in the other tissues (Fig. 3c, d). In the first offspring generation, *Sodalis* colonized the same tissues, and we additionally observed the sporadic presence of bacteria in the male testes, although the bacteria—when present—seemed to be less abundant compared to female ovaries (Supplementary Fig. 2). In contrast to the parental generation, *Sodalis* was more abundant in the bacteriomes, even though its density varied even within the organs—while some bacteriocytes harbored high loads of *Sodalis*, other bacteriocytes of the same bacteriome were apparently uninfected (Fig. 3g, h, and Supplementary Fig. 7). We could not detect bacteriomes and the native symbiont *Shikimatogenerans* in two of the twelve *Sodalis*-infected F1 beetles we sampled for microscopy (Supplementary Fig. 7). In the third offspring generation, all six *Sodalis*-infected specimens (five adults and one pupa) were devoid of the native symbiont (Fig. 3j, and Supplementary Fig. 8). The seeming absence of bacteriomes in the respective F1 and F3 individuals either indicates that the beetles fail to develop these organs following the loss of *Shikimatogenerans* or that we failed to detect empty and thus atrophied bacteriomes. In contrast to *Sodalis*-infected F3 individuals, control beetles of the third and subsequent offspring generations retained fully developed bacteriomes (Fig. 3i, and Supplementary Fig. 8). The observed loss of *Shikimatogenerans* in *Sodalis*-treated beetles compared to control beetles could not be explained by chance (Fisher’s exact test, *P* < 0.001).

### *Sodalis* affects *Shikimatogenerans* and bacteriome phenotype

**Fig. 4:**
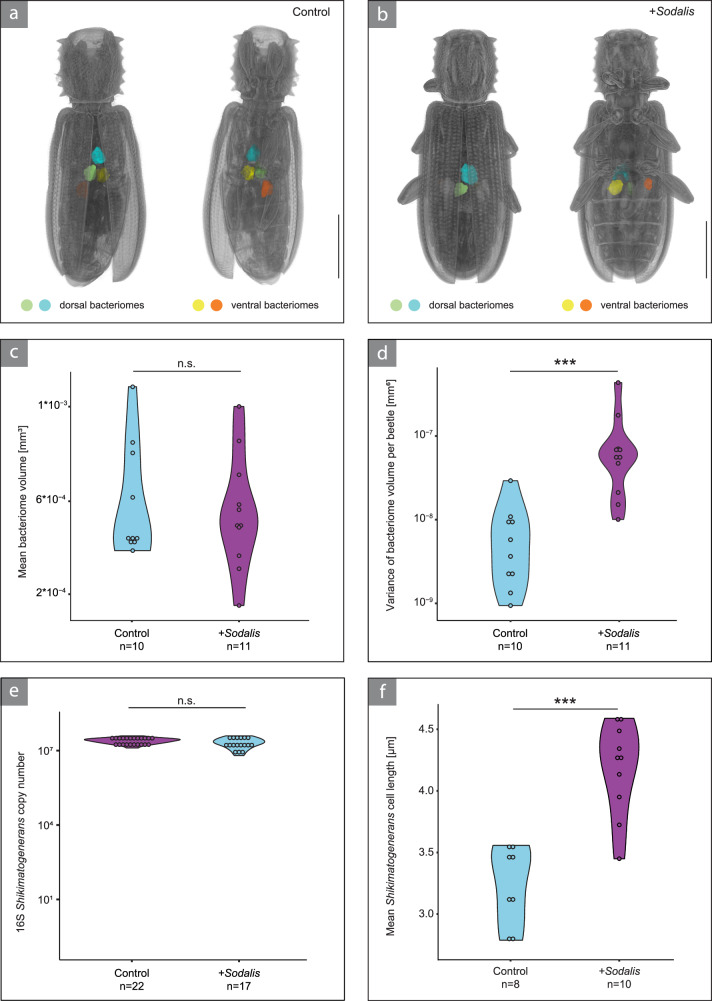
The presence of *Sodalis* affects bacteriome architecture and *Shikimatogenerans* cell size, but not *Shikimatogenerans* titer. 3D reconstruction of 7-day old adult F1 beetle offspring belonging to the control (**a**) and *Sodalis*-infected (**b**) treatment. Dorsal and ventral bacteriomes were reconstructed based on µCT scans and are labelled in blue/green and yellow/orange, respectively. Scale bar represents 1 mm. **c** The presence of *Sodalis* did not lead to a change in mean bacteriome volume (two-sided Wilcoxon rank sum test, *P* = 1.0). **d** Mean variance of bacteriome volume within a beetle was higher when beetles harbored *Sodalis* (two-sample *t*-test, *P* = 3.95 × 10^−5^). **e** 16S copy number of *Shikimatogenerans* did not differ between F1 beetles of the control and *Sodalis* infected treatment (two-sided Wilcoxon rank sum test, *P* = 0.19). **f** Upon colocalization of bacteriocytes with *Sodalis*, the mean length of *Shikimatogenerans* cells significantly increased (two-sample *t*-test, *P* = 3.23 × 10^−5^). Asterisks indicate statistical significance. (^***^P < 0.001). Source Data are provided as a Source Data file.

Given the loss of *Shikimatogenerans* and the apparent lack of bacteriomes in F3 adults following *Sodalis* infection, we further investigated the impact of *Sodalis* on bacteriome size and *Shikimatogenerans* phenotype. Using µCT scans, we examined the influence of *Sodalis* on the bacteriomes of F1 beetles. We found that the mean bacteriome volume did not differ between beetles harboring *Sodalis* and uninfected beetles (Wilcoxon rank sum test, *W* = 55, *P* = 1; Fig. 4a–c). However, we observed an increase in the variance of bacteriome volumes in beetles harboring *Sodalis*, with one bacteriome per pair (dorsal/ventral) being bigger than the other (two sample *t*-test, df = 19, *t* = −5.32, *P* < 0.001, Fig. 4d). Despite the increased variance in bacteriome volume, the titer of *Shikimatogenerans* in F1 adults was unaffected by the presence of *Sodalis* and did not differ from the titer we observed in control beetles (Wilcoxon rank sum test, *W* = 234, *p* = 0.19; Fig. 4e). We also did not observe differences in titers in the parental generation (0 dpi: Wilcoxon rank sum test, *W* = 119.5, *P* = 0.79; 7 dpi: *t*-test, df = 27, *t* = 0.57, *P* = 0.57; 14 dpi: Wilcoxon rank sum test, *W* = 142, P = 0.11, Supplementary Fig. 1). However, in bacteriocytes harboring abundant *Sodalis*, we observed altered morphologies of the beetles’ native symbiont *Shikimatogenerans*: Colocalization of the two microbial partners led to enlarged and irregularly shaped *Shikimatogenerans* cells as compared to globular or rod-shaped *Shikimatogenerans* in bacteriocytes of uninfected control beetles (Fig. 3g, h). Quantifying the effect of *Sodalis* on *Shikimatogenerans* cell length, we detected a significant increase in cell length of at least 30% in the presence of *Sodalis* (two-sample *t*-test, df = 16, *p* = −5.71, *P* < 0.001; Fig. 4f).

### The host beetle, but not *Shikimatogenerans*, transcriptionally responds to *Sodalis* infection

**Fig. 5:**
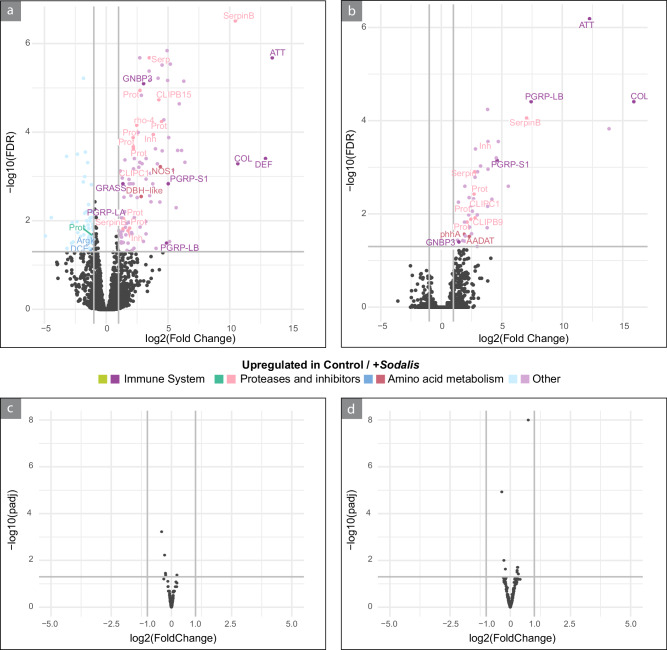
*Sodalis* infection significantly affects host but not *Shikimatogenerans* gene expression. Transcriptional responses of *O. surinamensis* and its ancient *Shikimatogenerans* symbiont to infection by *Sodalis* in the bacteriomes. Volcano plots of differential gene expression analysis results for *O. surinamensis* (**a**, **b**) and *Shikimatogenerans* (**c**, **d**) in the bacteriomes of the parental (**a,** **c**) and the first offspring generation (**b,** **d**), respectively. Lines represent the thresholds for gene expression regarding log2 fold change and -log10 of false discovery rate (**a**, **b**) or adjusted *p*-value (**c**, **d**, Wald test with Benjamini–Hochberg correction for multiple testing). Source Data are provided as a Source Data file.

To understand how the *Sodalis* infection affected host and symbiont transcriptional responses in the bacteriomes of *O. surinamensis*, we analyzed gene expression of *O. surinamensis* and *Shikimatogenerans* in the bacteriomes of adult *Sodalis*-injected versus control beetles, as well as their offspring 7 days after reaching adulthood. *O. surinamensis* showed a significant upregulation of many immune system genes in the presence of *Sodalis* (Fig. 5a, b, and Supplementary Fig. 9a). In both generations, the genes with the highest fold change difference and significance were immune genes such as antimicrobial peptides (AMPs), peptidoglycan recognition proteins LB (PGRP-LB) and S1 (PGRP-S1), serine protease inhibitors (serpins) and proteases. We also detected differentially expressed genes involved in amino acid metabolism: Dopamine beta-hydroxylase was upregulated in *Sodalis*-infected bacteriomes of the parental generation and followed the same albeit non-significant trend in the first offspring generation (Supplementary Fig. 9). Furthermore, a Kynurenine/alpha-aminoadipate aminotransferase (AADAT) and a phenylananine-4-hydroxylase (phhA) were upregulated in the F1 generation of *Sodalis*-infected beetles. No genes in *Shikimatogenerans* demonstrated differential expression in accordance with a definition of statistical significance involving at least a log2-fold change of normalized transcripts over |1| in *Shikimatogenerans* and a corrected *p*-value threshold of 0.05 (Fig. 5c, d). Yet, we note that the gene showing the highest level of differential expression encodes a proline dehydrogenase (PRODH), which was significantly differentially expressed with a log2-fold change of 0.75 (i.e., a 1.68-fold upregulation) in the F1 generation. Besides PRODH, we observed a total of 13 other genes to be significantly differentially expressed, albeit below a log2-fold change of 1 in the parental (upregulated: not annotated; downregulated: *groES, MTFMT, rsml*) and the F1 generation (upregulated: *mvaK1, rpoB, rpoC, sufB, sufC*; downregulated: *rpmg, prtC, recJ*). We also investigated the 100 most highly expressed protein-coding genes in *Sodalis* in both generations. In the parental generation, 20 of the 100 most highly expressed genes were predicted to be involved in the flagellar apparatus or its regulation (Supplementary Fig. 10a). By contrast, the respective motility genes were significantly downregulated in the first offspring generation (Supplementary Fig. 10b). We also detected genes of the type three secretion system (T3SS, e.g., *sseD*, *sseB*, *yscU* and *yscF*) and genes involved in quorum sensing (e.g., *expI*, *expR*, *hfq* and *secG*) to be highly expressed in both beetle generations (Supplementary Fig. 10a). Additionally, several genes encoding metabolism regulators, namely *ptsH*, *rpoH*, *crp* and *crr* were identified among the top 100 genes in both generations.

## Discussion

Intracellular symbioses with bacteria fundamentally impact insect ecology and evolution and can remain stable for hundreds of millions of years. Occasionally, however, even ancient and metabolically tightly integrated symbionts can be lost when their benefits deteriorate or become obsolete^10^ or when other microorganisms colonizing the host outcompete the native symbionts. In the latter case, the acquired microorganisms can functionally replace the original symbiont and/or provide additional benefits, occasionally expanding the host’s ecological niche space. Based on phylogenetic reconstructions, losses and replacements of ancient intracellular symbionts have been described across a variety of insect taxa, including spittlebugs, cicadas, aphids, and beetles^9–12,32–34^. The prerequisites for a symbiont replacement comprise (i) the establishment and persistence in a host, (ii) reliable vertical transmission, (iii) loss of the original symbiont, and (iv) a net benefit provided by the new symbiont. Yet the early steps and dynamics of these replacement events remain poorly understood, due to the lack of tractable study systems. Here, we describe the establishment of such a tractable symbiosis and report on the rapid loss of the host’s ancient beneficial symbiont upon establishment of a novel vertically transmitted bacterium. Our findings thus recapitulate the collapse of an ancient beneficial symbiosis and an important step towards a symbiont replacement, meeting three out of four necessary prerequisites, namely (i)–(iii), but not (iv).

To replace an ancient symbiont, an invading microbe like *Sodalis* must be capable of establishing in its new host by withstanding the insect’s immune system. Most intracellular beneficial symbionts likely evolved from pathogenic bacteria^2^ that employ two general strategies for coping with host immunity: (i) evasion of host immunity, i.e., preventing the host from mounting an immune response^35–37^, or (ii) resistance to host immune effectors^36^. Following infection with *Sodalis*, AMPs, PGRP-LB and other PGRPs, serpins, as well as other host immune effectors (e.g., dual oxidases (DUOX) producing reactive oxygen species, see Supplementary Fig. 9) were upregulated in the host bacteriome. Thus, the novel symbiont elicits an immune response but can persist despite the upregulation of host effectors, indicating resistance to host immunity. Indeed, the resistance of *S. praecaptivus* and its congener *S. glossinidius* to different AMPs has been demonstrated in vitro as well as in vivo and is mediated by the PhoP-PhoQ two-component system that regulates the expression of genes involved in modifying bacterial lipopolysaccharide (LPS) and thereby prevents damage by AMPs in vivo^38–40^. Apart from the upregulation of AMPs in *Sodalis*-infected bacteriomes, we also detected the upregulation of PGRP-LB and serpins in both investigated beetle generations. PGRP-LB is a negative regulator of the Imd immune pathway and plays a crucial role in the maintenance of the symbiosis between *Sitophilus zeamais* and *S. pierantonius*^41,42^. In the weevil bacteriomes, PGRP-LB is highly expressed and cleaves the peptidoglycan of *S. pierantonius* into non-immunogenic fragments, preventing an immune response that would lead to the elimination of the symbiont^42^. In contrast to *S. pierantonius*, *Shikimatogenerans* lacks a cell wall^29^—accordingly, PGRP-LB is not constitutively expressed in control *O. surinamensis* bacteriomes (Supplementary Fig. 9b) but might rather regulate host immunity after pathogen infection. The upregulation of PGRP-LB and serpins in the presence of *Sodalis* might modulate the immune response to prevent a negative impact of immunity on the host or damage to the native symbiont *Shikimatogenerans* by the beetle’s immune effectors. Despite the elevated expression of negative regulators of the host immune response, we observed a significant upregulation of effectors involved in the Imd pathway, likely incurring a cost on the insect host and potentially causing or contributing to the observed detrimental impacts on the native *Shikimatogenerans* symbiont.

After successful establishment, newly acquired symbionts need to secure their continuous association with the host by achieving efficient transmission^43^. Although not native to *O. surinamensis*, *Sodalis* readily invaded the host’s germline and infected the developing eggs, thereby establishing a highly efficient transovarial vertical transmission route. *Sodalis’* motility genes were among the most highly expressed genes in bacteriomes of newly infected beetles, but not in the following generation, suggesting a role of motility in the initial infection, as seen in other systems^44–46^. Additionally, successful colonization and transmission likely depend on *Sodalis’* T3SS that allows for the invasion of host cells in bacterial pathogens and some beneficial symbionts^47–49^. Indeed, genes encoding for T3SS components (e.g., *sseD*, *sseB*, *yscU* and *yscF*) were among the most highly expressed genes in *Sodalis* in both P and F1 beetle generations (Supplementary Fig. 10a). Our study provides further support for the notion that efficient vertical transmission can occur spontaneously in new host-symbiont combinations. However, it also demonstrates that there is a strong host component contributing to transmission, since the same *Sodalis* strain is transmitted at lower efficiency in another beetle host, *S. zeamais*, that natively harbors a *Sodalis* symbiont^19^, and fails to achieve any vertical transmission following injection into *Drosophila melanogaster*^50^.

In insects that already harbor intracellular symbionts, newly acquired vertically transmitted microbes can coinhabit their new niche with the host’s native symbionts, providing opportunities for symbiont-symbiont interactions that can determine the outcome of the coinfection. Although symbiont replacements are widespread across insects, little is known about the initial interactions between new and native symbiont. Limited host resources could drive microbial competition for nutrients, which might disrupt the tightly regulated metabolite exchange between native symbiont and host and could ultimately lead to the exclusion of one microbial partner, as observed in this study. Nutritional competition among intracellular bacteria residing in the same host is an understudied phenomenon, especially in the context of symbiont replacements. Yet, metabolic models predict that obligate *Portiera aleyrodidarum* and facultative *Hamiltonella defensa* symbionts compete for nutrients supplied by their whitefly (*Bemisia tabaci*) host, thereby limiting *Portiera* growth and resulting in a deficit of nutrients supplied to the insect^51^. Similarly, competition for nutrients is suggested to explain the negative effect of *Spiroplasma* or *Serratia symbiotica* on the abundance of *Wolbachia pipientis* in *D. melanogaster*^52^ or *Buchnera aphidicola* in *Acyrthosiphon pisum*^53^, respectively.

Since competition for resources among coinfecting microbes seems to occur across different insect systems, it may also explain the rapid loss of *Shikimatogenerans* upon introduction of *Sodalis*: The native symbiont *Shikimatogenerans* aids its beetle host by supplying it with prephenate, a precursor of tyrosine needed for robust cuticle synthesis and melanization^29^. Due to a highly eroded genome, the symbiont is not able to produce any other nutrients, such as vitamins, amino acids, fatty acids or nucleotides^29^. Thus, the host needs to supply these metabolites to the symbiont to enable the biosynthesis of prephenate. The bacteriomes are therefore expected to contain high concentrations of host-provided nutrients that could be prone to exploitation by bacteria like *Sodalis* that invade the symbiont-bearing organs. Exemplary for the exploitation of host-supplied nutrients is the depletion of tyrosine by *Sodalis*, as evidenced by a decrease in beetle melanization and the upregulation of host *phhA* in the bacteriomes, mediating the conversion of phenylalanine into tyrosine. Similarly, AADAT expression was upregulated in bacteriomes following *Sodalis* infection. Analogous to a glutamate oxaloacetate transaminase in *Rhynchophorus ferrugineus* weevils^54^, promiscuous AADAT^55^ can be involved in converting prephenate and its derivative 4-hydroxyphenylpyruvate into tyrosine. These findings are indicative of a tyrosine starvation response in the beetle^56^ and suggest the disruption of host-symbiont metabolite exchange via nutritional competition between *Sodalis* and *Shikimatogenerans*.

Apart from the host, we also detected signs of nutrient depletion in the ancient symbiont. Interestingly, in *Shikimatogenerans*, the most highly upregulated transcript encodes a PRODH (log2-fold change = 0.75), that is involved in proline catabolism leading to the production of glutamate which can serve as a donor of amino groups or be used for protein biosynthesis. Upregulation of PRODH is therefore consistent with amino acid starvation and corroborates the hypothesis that *Sodalis* infection disturbs the metabolic exchange between host and native symbiont. Beyond PRODH, however, our RNAseq data indicate that *Shikimatogenerans* appears to be limited in its ability to modulate its gene expression. Like many other ancient and genome-eroded insect endosymbionts, the *Shikimatogenerans* gene inventory lacks protein-coding genes that are predicted to serve as transcriptional regulators (but see Carvalho et al.^57^). Yet, we note that transcription could be modulated through other means such as non-coding small RNAs and interactions between metabolites and transcription machinery.

Facilitated by the symbiont’s reduced capacity to modulate its gene expression and respond to stressors, as well as the absence of a bacterial cell wall to protect cell integrity^58^, changes in the nutritional and/or physiochemical environment in the bacteriome caused by *Sodalis* (and potentially exacerbated by the host immune response) could result in the observed morphological aberrations and the ultimate elimination of the native symbiont. Still, the outcome of symbiont-symbiont interactions seems highly dependent on the combination of partners. While in *O. surinamensis*, *Sodalis* infection causes the rapid loss of *Shikimatogenerans*, the same strain failed to stably coexist with the more recently acquired congener *S. pierantonius* in *S. zeamais* due to reduced transmission efficiency in the presence of the native weevil symbiont^19^.

While the introduction of *Sodalis* into *Oryzaephilus* beetles meets three out of four necessary prerequisites for a symbiont replacement, *Sodalis* did not provide a net benefit to its novel host and, thus, remained pathogenic in our experimental setup. Generally, it is assumed that ancient symbionts are replaced because the benefits they provide deteriorate over time due to genome erosion, resulting in selective pressures on the host to favor symbionts that provide the benefits more efficiently^59^. Thus, when a novel symbiont replaces its host’s ancient symbiont, it is commonly expected that it will evolve necessary beneficial properties before the native obligate symbiont is eliminated. Still, under certain conditions, hosts might be able to tolerate the loss of an ancient symbiont before establishing a mutually beneficial relationship with another microbe. This particularly applies to cases where the novel microbe is the main driver of the native symbiont’s elimination and where the ancient symbiont is not essential for host survival and provides context-dependent benefits to the host. While novel microbes as drivers of symbiont losses have not previously been described in the literature, context-dependent symbiont benefits are common in insect-microbe symbioses^6,25,60–63^. This also applies to the *Oryzaephilus* system, where beetle hosts can survive without *Shikimatogenerans*, at least under conditions of low desiccation stress and antagonist pressure. We hypothesize that *Sodalis* could be capable of evolving a benefit to the host and functionally replacing the native symbiont, based on several factors: First, *Sodalis* should experience high selection pressure to attenuate its virulence and reduce the negative impact on host fitness due to its strictly vertical transmission route. The invasion of reproductive tissues and the ability to achieve vertical transmission are important steps towards evolving a mutualistic relationship with a host, since vertical transmission aligns host and symbiont fitness and thereby selects for symbionts with host-beneficial phenotypes^30,31^. As such, vertical transmission is generally thought to be a key driver in the evolution of beneficial intracellular symbioses^2^. Second, given its metabolic potential, it is conceivable that *S. praecaptivus* could evolve a beneficial phenotype, e.g., via leakage of metabolites like essential amino acids or vitamins that can lead to the establishment of beneficial cross-feeding in bacteria-bacteria^7,64^ and bacteria-eukaryote interactions^65^. Concordantly, previous studies revealed that mutualisms can evolve rapidly, as observed in *Escherichia coli* where single mutations in either *cyaA* or *crp*, two genes involved in carbon catabolite repression (CRR), were sufficient to render it a mutualist of stink bugs^66^. Interestingly, *crp* as well as *crr* and *ptsH*, which are also involved in CRR, are among the 100 most highly expressed genes of *Sodalis* in the beetle host and could be potential targets for experimentally manipulating *Sodalis* to become more benign. Third, the potential of *Sodalis* to form lasting beneficial symbioses is supported by the high number of *Sodalis*-allied symbionts in various insect taxa^67–71^. While functionally replacing a highly efficient and streamlined ancient symbiont is not trivial, many *Sodalis* symbionts have apparently succeeded in substituting other intracellular insect symbionts^11,34^. The success of this scenario, however, will highly depend on the speed at which virulence attenuation can be achieved and beneficial traits evolve.

In conclusion, the elimination of *Shikimatogenerans* following the introduction of *Sodalis* in *O. surinamensis* showcases the fragility of ancient intracellular symbioses and reveals that symbiont losses can occur rapidly upon acquisition of a novel symbiont. We experimentally demonstrate that newly acquired microbes can drive losses of ancient intracellular symbionts, potentially leading to the replacement of non-obligate or context-dependent host-beneficial microbes. Given that *Sodalis* is genetically tractable, the *Oryzaephilus*-*Sodalis* symbiosis allows for the investigation of symbiont elimination and replacement as well as the transition from pathogenicity to mutualism on a molecular level. Such experimentally and genetically tractable systems are urgently needed to deepen our understanding of the establishment and maintenance of animal-microbe symbioses and to study the evolution of host-beneficial interactions in real time.

## Methods

### Insect cultures and bacterial strains

*O. surinamensis* beetles, harboring the intracellular symbiont *S. silvanidophilus*, were originally provided by the Julius-Kühn-Institute/Federal Research Centre for Cultivated Plants (Berlin, Germany) in 2014. Since then, these beetles have been reared on organic oat flakes (Huber Mühle, Germany) under controlled conditions of 27–28 °C and 60% relative humidity (rH) following a day/night cycle of 16/8 h, respectively.

As a culturable symbiont, we used a strain of *S. praecaptivus* (strain MC1, identifier: CD2555, Su et al.^19^) that expresses fluorescent mCherry protein, enabling us to observe infection of beetles in vivo, since infected beetles exhibit strong fluorescence.

### Establishing *Sodalis* in *O. surinamensis*

*Sodalis* was introduced into *O. surinamensis* by microinjecting a bacterial suspension into the hemolymph of female adults, following established protocols^47^. Briefly, we grew *Sodalis* in liquid LB medium overnight, at 28 °C and 220 rpm. After incubation, we concentrated bacteria to OD_600_ = 1.0 in physiological saline (0.85% NaCl (w/v)) under sterile conditions. Using a Flaming/Brown micropipette puller (Model P-1000, Sutter Instruments, USA), we pulled needles from 3.5” glass capillaries (#3-000-203-G/X, Drummond, USA) according to the following settings: heat=512, pull=45, velocity=75, delay=90, pressure=500. Oil-filled needles were mounted to a Nanoject III microinjector (Drummond, USA) and loaded with bacterial suspension. 10 nl of suspension with OD_600_ = 1 (translating to 6.01 × 10^3^ ± 2.79 × 10^3^ CFUs) was injected into the abdomen of fully melanized *O. surinamensis* females at a speed of 10 nl/s. Beetles of the control treatment were injected with physiological saline. To facilitate injections, we cut off the posterior third of the beetles’ elytra the day before the injections and immobilized the insects on a cold metal plate during the procedure. Injected females were of undefined age. However, since they were fully melanized, we considered them to be at least 2 weeks old and therefore at a reproductive age^72^.

After the injections, we transferred each beetle into a fluon-lined well of a 48-well plate (Greiner Bio-One, Germany) filled with oat flakes and incubated the plates at 27–28 °C and 60% rH. Injections were performed in seven batches with 50 beetles per treatment. Two specimens per batch were immediately stored in ethanol for qPCR (see “qPCR” section).

To assess the establishment of *Sodalis* in *O. surinamensis*, we inspected live female beetles for mCherry fluorescence using a Leica M165FC fluorescence stereomicroscope (Wetzlar, Germany) 7 days after injections were performed.

### Monitoring survival and fitness correlates after *Sodalis* infection

To investigate the influence of *Sodalis* on host survival, we monitored the survival of *Sodalis*-injected and control beetles for a period of 80 days. Beetle survival was monitored every 24 h for the first 3 weeks and twice a week thereafter. 0, 7, and 14 days post injection (dpi), a subset of infected females was processed for additional analyses, see the following sections for more details on the sampling process.

During the observation period, we counted the number of first offspring generation (F1) larvae produced by each injected female as a proxy for fitness. We checked for the presence of larvae on a daily basis for the first 3 weeks, and once a week thereafter. When we found larvae in a well, each larva was carefully moved to a fluon-lined well of a new 48-well plate using a toothpick. Survival and development of isolated larvae were monitored every 2 days, and we switched to daily observations once the larvae neared pupation to be able to assess the exact date of adult emergence. After 28 days, we stopped moving larvae of the control treatment to new wells, since we had already collected enough for the coming sampling. Instead of moving the larvae, we now transferred the female beetles to new plates, leaving the larvae in the wells. We counted larvae on the day of transfer and 10 days after, to account for individuals that might have hatched from eggs after the transfer of the adult female. For both treatments, we let isolated F1 larvae develop into adults, recording the day adults emerged from the pupa. Seven days after eclosion, we fixated F1 adults for further analyses. For more details, see the following sections. For all analyses concerning the F1 offspring of *Sodalis-*treated beetles, we only included individuals that were confirmed to harbor *Sodalis* based on mCherry fluorescence observed under a Leica M165FC fluorescence stereomicroscope.

We transferred excess F1 beetles of both treatments that were not needed for further analyses to small plastic boxes, supplied with oats. Offspring of these beetles were transferred to a new box, where they developed into F2 adults and reproduced. F3 offspring larvae were transferred into a 48-well plate, where they developed into the pupal stage and subsequently adulthood. The few surviving *Sodalis*-infected F3 beetles (five adults and one pupa) were fixated for FISH to investigate the localization of *Sodalis* and its impact on *Shikimatogenerans*. Uninfected controls showed much higher survival, so only a subset of the beetles was maintained. To confirm the long-term maintenance of *Shikimatogenerans* in control beetles, individual beetles from approximately generations 8-10 were fixated for FISH.

### *Sodalis* transmission efficiency experiments

Late instar larvae and pupae were separated from the main *O. surinamensis* lab colony and reared individually in fluon-lined wells of a 48-well plate supplied with oat flakes until adults emerged. A subset of virgin female and male adults was injected with 10 nl of *Sodalis* suspended in 0.85% NaCl solution (OD_600_ = 1.0) and kept in individual wells for 1 week to let beetles recover from the injection and wait for *Sodalis* to establish. After a week, we checked for the presence of mCherry fluorescence, indicative of *Sodalis* infection, in the injected beetles. Using infected beetles, two mating groups were set up to assess maternal and paternal *Sodalis* transmission, respectively. We placed one male and one female beetle in each well of a 24-well plate (Greiner Bio-One, Germany)—*Sodalis* infected females were placed with uninfected males, and uninfected females were placed in wells with *Sodalis* infected males. The mating pairs were kept in the wells for 21 days, after which we checked both adult beetles for *Sodalis* presence (i.e., mCherry fluorescence) to see if bacteria were transmitted horizontally between individuals. At the same time, we separated the produced larvae from the parents and screened for *Sodalis* by fluorescence stereomicroscopy. Using the number of infected and non-infected larval offspring, we calculated the mean transmission efficiency of *Sodalis* for the two groups.

### Examining symbiont titers via qPCR

To study the bacterial load and its dynamics in *O. surinamensis*, we sampled beetles of the parental generation and F1 adults. Immediately after injection, 7 and 14 dpi, adults of the parental generation were fixed in 70% ethanol and stored at −20 °C until DNA extraction. For the F1 offspring, we sampled adults 7 days after eclosion. Prior to DNA extraction, samples were placed in individual Eppendorf tubes, and the remaining ethanol was evaporated in a vacuum concentrator (Concentrator 5301, Eppendorf, Germany). We extracted DNA of individual beetles using the Epicentre MasterPure complete DNA and RNA purification kit (Lucigen, USA) according to the manufacturer’s instructions. DNA was eluted in 30 µl of low TE buffer.

For qPCR, we combined 19 µl of mastermix (10 µl Blue S’Green qPCR mix (Biozym, Germany), 7.4 µl nuclease-free water, 0.8 µl of each primer) with 1 µl of template DNA in the wells of a 96-well plate (Bio-Rad, USA). We amplified all samples on a CFX Connect cycler (Bio-Rad, USA) according to the following conditions: initial denaturation for 15 min at 95 °C, 60 cycles 95 °C for 15 s, 60 °C for 30 s and 72 °C for 30 s followed by a final denaturation step at 95 °C for 15 s. Then a melting curve was recorded by incrementing the temperature by 0.5 °C every 5 s from 55 °C up to 95 °C. To allow for absolute quantification of input material, we included a set of standards ranging from 1 to 10^−7^ ng/µl of amplicon on each plate. Using the standards, the qPCR program (CFX Manager v3.1, Bio-Rad, USA) assessed the starting quantities of the respective fragment in our samples, from which we calculated the corresponding copy number. To amplify the 16S gene of *Shikimatogenerans* we used the OsurSym_fwd2 (5′-GGCAACTCTGAACTAGCTACGC-3′) and mod. CFB563_rev (5′-GCACCCTTTAAACCCAAT-3′) primers^6^. For *Sodalis*, we amplified part of the *tam* gene using the primers SP_tam3_fwd (5′-CGTCTTAGCGGTACAAATGCC-3′) and SP_tam3_reverse (5′-GCAGATCGTAAT-AGTCGGCG-3′) modified from Medina Munoz et al.^73^.

### Melanization

For the F1 offspring, we analyzed cuticle coloration as a measurement of melanization that may reflect tyrosine availability to the host^6^. We collected F1 adults 7 days after eclosion and immobilized them on a cold metal plate. Using a Leica M165 FC stereomicroscope (Leica, Germany), we obtained pictures of the beetles with standardized settings and illumination conditions (2× magnification, 293,5 ms exposure time, 50 gain, 6.2 MP resolution) that we used to analyze the color of beetle thoraces and abdomens. Pictures were imported into the Natsumushi software v1.10^74^, and average red values were extracted from circular areas for both thorax (radius = 326.7 pixels) and abdomen (radius = 388.9 pixels). Average red values were transformed to inverse red values and compared between treatments. Specimens photographed for the assessment of melanization were then fixated for cuticle thickness measurements.

### Cuticle thickness

Besides melanization, we also examined the cuticle thickness of F1 adults as a proxy of tyrosine availability. Cuticle thickness was assessed using micro-computed tomography (µCT) scans of adult F1 beetles. To this end, we fixated 7 d old adults in 4% paraformaldehyde (PFA) in 80% ethanol and removed their heads to facilitate entry of the fixative into the body cavity. Samples were stored in fixative at 4 °C until we prepared them for µCT according to Janke et al.^75^. Briefly, we transferred beetles to absolute methanol to wash out the fixative and incubated the beetles for 24 h. Afterwards, beetles were moved to 1% iodine in absolute methanol and incubated at room temperature for another 24 h to enhance the contrast of internal tissues. Beetle specimens were then washed in absolute ethanol (denatured) for 1 h on a shaker (Unimax1010, Heidolph, Germany) to remove excess iodine. The washing step was repeated twice, followed by 3 washing steps in 100% absolute ethanol on a shaker for 1 h each. Washed beetles were dried in a critical point drier (Leica EM CPD300, Leica, Germany) with medium CO_2_ supply with a delay of 20 min, 20 exchange cycles, heating at medium speed and slow gas exhaust. Dried specimens were mounted in translucent 10 µl pipette tips with pipette tip pieces as spacers in between samples.

Pipette tips with samples were placed on the sample holder and scanned in an X-ray microtomograph (Skyscan 1272, Bruker, USA). We created 360° scans with 0.2° rotation steps, the voltage and electric current were adjusted to 35–40 kV and 135–200 mA, respectively, to achieve a signal attenuation of 35%. Final pixel size of the scans was 2 µm with a resolution of 2452 × 1640.

Based on the scans, we performed 3D reconstruction of the scanned specimens using the NRecon software (v2.1.0.1, Bruker, USA) with the following settings: ring artefacts reduction = 10 and beam hardening correction = 20%. 3D reconstructions were imported into Dragonfly^76^ (v2022.2, Comet Technologies Canada Inc., Montreal, Canada; software available at https://www.theobjects.com/dragonfly) where we measured beetle cuticle thickness in two ways: First, we measured mean cuticle thickness across the whole insect’s body (excluding the head, which had been removed to facilitate fixation), by extracting the cuticle from the 3D reconstruction and applying a thickness mesh. The mesh was then used to extract mean cuticle thickness. Additionally, we measured the mean cuticle thickness in a single section of the thorax, between the 3rd and 4th lateral spike. Thickness was measured in a dorsal, ventral and sagittal position of the section and the individual measurements were averaged.

### Symbiont localization via fluorescence-in-situ-hybridization (FISH)

To examine localization of *Sodalis* in the beetles, we fixated adult beetles of the parental generation 7 dpi and of the F1 generation 7 days after eclosion in 4% PFA in 80% butanol for FISH. Sample preparation was conducted according to Weiss^77^: The extremities of fixated beetles were removed to facilitate fixation of the remaining tissues, and samples were pre-embedded in 1% agar blocks. Beetle specimens were then washed in 80% tertiary butanol and subsequently dehydrated in a series of butanols with ascending concentrations (90%, 96% and 100%). After a final dehydration step in acetone, samples were infiltrated with, and finally embedded in, HistoCURE 8100 (Morphisto, Germany) according to the manufacturer’s instructions. Embedded beetles were cut into 8 µm thin sagittal sections with a Leica Histocore AUTOCUT microtome equipped with glass knives. Sections were placed on silanized glass slides that were covered with 100 µl of hybridization mix consisting of hybridization buffer (0.9 M NaCl, 0.02 M Tris/HCl, pH 8.0, 0.01% SDS), 0.5 µM fluorescently labeled oligonucleotide probes and 0.5 mg/ml 4′,6-diamidino-2-phenylindole (DAPI). As probes, we used one probe specific for *Shikimatogenerans* (OsurSym_16S_Cy5, 5′-AGCAGCCCATTGGACTAACC-3′), one specific for *Sodalis* (Sod_FISH-Cy3, 5′-TCCGCTGACTCTCGCGAGAT-3′; Shalom et al.^78^), and a general probe targeting eubacteria (EUB338-RhodamineGreen, 5′-GCTGCCTCCCGTAGGAGT-3′; Amann et al.^79^). We applied cover glasses and incubated the slides in a humid chamber at 50 °C overnight. The next day, we removed the cover glasses, transferred the slides to wash buffer (0.1 M NaCl, 0.02 Tris/HCl, 5 mM EDTA, 0.01% SDS) and incubated them at 50 °C for 2 h. Afterwards, we washed the slides in distilled water for 20 min and mounted them with Vectashield (VectorLabs, USA). Sections were imaged with a Leica THUNDER imager DMi8 equipped with a DFC9000GT camera and CYR71010 and DFT51010 filter cubes. Pictures were processed with small volume computational clearing in the Leica Application Suite X software (Version 3.8.2.27713, Leica, Germany). Besides beetles of the parental and F1 generations, we also performed FISH on individuals of F3 and the following generations.

### Measuring the impact of *Sodalis* on *Shikimatogenerans* cell length and bacteriome volume

In each beetle sample used for FISH, we assessed the effect of *Sodalis* on the cell size of *Shikimatogenerans*. To provide a conservative estimate of this effect, we pre-selected *Shikimatogenerans* cells that resembled the shape of the symbiont in the control treatment the most. Among these selected cells, we measured the length of 10 random *Shikimatogenerans* cells in one picture of a random bacteriome. The size of 10 cells was averaged per sample and then used to compare cell sizes between the two treatments. Besides *Shikimatogenerans* cell length, we also investigated the impact of *Sodalis* on bacteriome volume. Using Dragonfly (v2022.2), we marked the bacteriomes in each section as a region of interest (ROI) and interpolated between the sections to obtain the entire bacteriome volume. We manually corrected ROIs to only cover the bacteriomes and none of the surrounding tissues. For each of the bacteriomes, we extracted the number of voxels each ROI covered and calculated the total volume of each bacteriome accordingly (1 voxel = pixelsize^3^).

### Gene expression in the bacteriome: RNASeq analysis of *O. surinamensis*, *Shikimatogenerans* and *Sodalis*

#### Sample collection, RNA extracting, and sequencing

To investigate gene expression of *Shikimatogenerans, Sodalis* and *O. surinamensis*, we dissected bacteriomes of adults of the parental generation 7 days after injection, and of F1 adults 7 days after eclosion. Both control and *Sodalis* infected beetles were dissected in sterile 1.5× phosphate-buffered saline, and bacteriomes were transferred into a drop of Trizol in the lid of a reaction tube. Per treatment group, we dissected 7 replicates of pooled bacteriomes, making sure that for each pool, at least 15 bacteriomes were collected (out of 20 possible for the five beetles, since there are four bacteriomes in each individual beetle). For the parental generation, we pooled the bacteriomes of 5 beetles in 100 µl of Trizol and added 150 µl Trizol before RNA extraction, while for the F1, we collected bacteriomes of individual beetles in 50 µl of Trizol and pooled them just before extracting RNA. Bacteriomes in Trizol were manually homogenized using tweezers and collected at the bottom of the tube by spinning down the Trizol. Bacteriomes were stored at −80 °C until RNA extractions. Tweezers were cleaned with 70% ethanol in between dissections, and the buffer was changed between samples. RNA was extracted using the Directzol RNA Microprep kit (Zymo Research, USA) according to the manufacturer’s instructions. We eluted RNA in 15 µl of nuclease-free water and stored it at −80 °C until sequencing. Following the extraction, RNA quality and quantity were assessed by capillary electrophoresis using an Agilent Bioanalyzer PicoChip (Agilent, USA). A total RNA library was prepared with a Zymo-Seq RiboFree Total RNA Library kit (Zymo Research, USA) as outlined in the manufacturer’s instructions. Total RNA inputs ranged from 7 to 15 ng and library preparation was adjusted according to the protocol recommendations for low input amounts. Library fragments passing the universal depletion step were then amplified by PCR to convert single-stranded cDNAs into double-stranded cDNAs. We sequenced 2 × 150 bp paired reads on an Illumina NextSeq 2000 sequencer with a depth of approximately 50,000,000 reads per library. RNA quality control, rRNA depletion, library preparation and sequencing were performed at the Max Planck Genome Center in Cologne.

#### Read processing and mapping

For the analysis of *Sodalis* and *Shikimatogenerans* gene expression, all reads were trimmed using Trim Galore v0.6.10^80^ for a Phred quality score threshold of 30 and minimum read length of 50 bp. Potential remaining rRNA reads were removed using Sortmerna v4.3.6^81^ against the default database smr_v4.3_default_db. Of the on average 93.7 million total reads, an average of 82.8 million reads were retained after trimming and rRNA removal and used in the subsequent analysis. For the host gene expression analysis, RNASeq reads were trimmed, mapped and counted against the *O. surinamensis* genome using the NF-CORE/RNASeq pipeline v3.18^82^ with the trimgalore, remove_ribo_rna and star_salmon options.

The published genomes of *S. silvanidophilus* (GCF_018200315.1) and *S. praecaptivus* (GCA_000517425.1) were obtained, and genes were predicted and annotated using Bakta v1.9.3^83^ with default parameters. The translated CDSs were then used to obtain KEGG functional annotations^84^ using KEGGREST v1.40.1^85^ in R v4.3.3^86^. Reads were mapped using Bowtie2 v2.4.1^87^ from end to end (sensitive mode), allowing for no discordant or mixed reads. The BAM files were sorted and indexed using Samtools version 1.13^88^. On average, 43.9% of reads mapped to the genome of *Shikimatogenerans*, and 0.37% of reads mapped to the genome of *Sodalis* (averaged across infected samples, control samples had 0.00% mapping). The aligned reads were counted with FADU v1.8.3^89^ using the GFF files from the above-mentioned genomes for gene features.

The *O. surinamensis* genome was assembled using previously published single-molecule long reads (SRR12881567–SRR12881568) obtained by sequencing genomic DNA on an Oxford Nanopore (ONT) MinION Mk1B sequencer. The ONT raw data were base-called using GUPPY v4.0.11^90^ with a high-accuracy option (dna_r9.4.1_450bps_hac.cfg model), generating 11.7 Gb of sequence reads with an N50 of 24.5 kb. Genome assembly was performed using Flye v2.9.2^91^ in meta-mode, followed by four iterations of polishing using Racon v1.4.13^92^ and one round of error correction using Medaka v1.0.3 (https://nanoporetech.github.io/medaka). For final error correction, we used ntEdit v1.3.2^93^ with paired-end (2 × 150 bp) Illumina data generated from the same DNA used for ONT sequencing. PurgeDups was used to remove duplications (heterozygous regions) and generate a haploid genome for downstream analysis. The relative contig coverage, GC content and contig taxonomic classification were scanned after genome assembly using BlobTools v1.0 and Taxon Analysis^94^ to enable the identification and removal of potential microbial symbionts or contaminants. The completeness of the genome was assessed using BUSCO v5.2.1^95^, utilizing the insect_odb10 database. The assembled haploid genome contained 1770 contigs with a total genome size of 142 Mb and an N50 contig size of 1,5 Mb. A genome completeness assessment using BUSCO resulted in 99.3% BUSCO complete and single-copy orthologs and low duplication levels (0.4% duplicated BUSCO). Genome annotation of *O. surinamensis* was performed using a combination of RNASeq data obtained from beetle bacteriomes (RNA extraction with Zymo Direct-zol RNA Miniprep kit, rRNA depletion and poly(A)-enrichment, 2 × 150 bp paired-end sequencing with a depth of 25,000,000 reads on a NextSeq 2000 device) and the reference protein database Arthropoda ODB10. Briefly, reads were trimmed using TrimGalore v0.6.10^80^ and read length and quality thresholds set as 50 bp and 30, respectively. Reads were then mapped against the *O. surinamensis* genome using HiSAT2 (v2.2.1) with default parameters except for the intron length which was set to 10 kb and the “dta” option. Mapped samples were then merged, and the resulting file was processed with samtools v1.17^88^ using the subcommands “collate”, “fixmate”, “sort” and “markdup” with default parameters. The final BAM file, along with the protein reference database, was then provided to BRAKER v3.0.7^96–104^ to perform gene model prediction. BRAKER predictions were post-processed to fix overlapping genes and retain only the longest isoform using AGAT v1.5.1^105^. Prior to annotation, the genome of *O. surinamensis* was softmasked using the RepeatMasker suite. A first round of masking using RepeatMasker v4.1.5^106^ was performed based on the insect lineage and the default database of RepeatMasker. In parallel, de novo prediction of repeats was performed using RepeatModeler v2.0.4^107^ with default parameters. A second round of softmasking was then performed using the custom repeats library produced by RepeatModeler.

Finally, nucleotide coding sequences and proteins were extracted with GFFREAD v0.12.8^108^. Functional information was obtained EggNOG (eggNOG-Mapper 2.1.0 with EggNOG 5.0.2, Huerta-Cepas et al.^109^) within OmicsBox v3.4.1 and InterProScan^110^.

#### Differential gene expression analysis

Gene expression was compared between control and *Sodalis*-infected beetles for each generation, except for *Sodalis*, in which gene expression was compared across generations for the *Sodalis*-infected treatments. For the host gene expression analysis, counting matrices were then subjected to differential expression analysis using EdgeR v4.2.2^111^ in R v4.4.0 using a quasi-likelihood test and an FDR threshold of 0.05 and a log2FoldChange of ≥|1|. Prior to differential expression estimation, genes with low variation were removed through the filterByExpr function of the EdgeR package. Out of the total 13,283 genes, 7811 and 7689 were kept in the analysis of the parental and first generations, respectively. A total of 150 and 46 genes were identified as differentially expressed in the parental and first generations, respectively.

For the bacterial gene expression analysis, differential gene expression was assessed using DESeq2 v1.40.2^112^ in R version 4.3.3^113^. Genes with fewer than a total of 20 reads across samples were excluded from the analysis. For *Shikimatogenerans*, 276 protein-coding genes were analyzed, whereas for Sodalis 4362 out of 4657 total genes were kept. For all differential gene expression analyses, the function lfcshrink was run using the adaptive Student’s *t* prior shrinkage estimator from the package apeglm v1.22.1^114^. Genes were considered differentially expressed if the adjusted *p*-value was ≤0.05 and the log2FoldChange was ≥|1|. No genes were identified as differentially expressed for *Shikimatogenerans*. Conversely, *Sodalis* differentially expressed 168 genes across generations. Additionally, *Sodalis* genes were sorted according to the average TPM values obtained from the FADU outputs for each generation, and the top 100 genes were selected out of the 2946 protein-coding genes annotated with BlastKoala v3.0.

### Statistics

We analyzed the survival of injected beetles with cox mixed-effect models v2.2.20 (coxme package^115^). Our models included treatment as an explanatory variable while the injection batch was included as a random factor. An effect of treatment was tested using a type II analysis of variance (ANOVA, car package v3.1.2^116^). The number of larvae produced by injected females was analyzed with a zero-inflated negative binomial model (pscl package v1.5.9^117^) using treatment as an explanatory variable. Bacteriome absence and presence in F3 individuals was compared between the two treatments using a Fisher’s exact test (stats package v4.2.3^86^). Remaining fitness parameters were compared between the two treatments using two-sided parametric two sample *t*-tests or non-parametric Wilcoxon rank sum tests (stats package v4.2.3^86^). Assumptions of normality and homogeneity of variance were verified using the Shapiro–Wilk test and Levene’s test, respectively (stats package v4.2.3^86^). Figures for survival and fitness parameters were created with ggplot2 v3.5.1^118^.

### Reporting summary

Further information on research design is available in the Nature Portfolio Reporting Summary linked to this article.

## Supplementary information

Supplementary Information

Reporting Summary

Peer Review file

## Source data

Source Data

## Data Availability

The annotated beetle and symbiont genomes and RNAseq data are available in the Edmond open data repository of the Max Planck Society under 10.17617/3.MUV1MF. RNAseq data generated in this study has also been deposited in the NCBI Sequence Read Archive under accession number PRJNA1423255 [http://www.ncbi.nlm.nih.gov/bioproject/1423255]. The bacterial genomes used in this study were obtained from NCBI using accession numbers GCF_018200315.1 [https://www.ncbi.nlm.nih.gov/datasets/genome/GCF_018200315.1/] (*Shikimatogenerans silvanidophilus*) and GCA_000517425.1 [https://www.ncbi.nlm.nih.gov/datasets/genome/GCF_000517425.1/] (*Sodalis praecaptivus*). Single-molecule long reads for assembling the *Oryzaephilus surinamensis* genome were obtained from the NCBI Sequence Read Archive under accession numbers [SRR12881567]-[SRR12881568]. RNAseq data used for annotation of the *Oryzaephilus surinamensis* genome is available in the NCBI Sequence Read Archive under accession number PRJNA1423469 [http://www.ncbi.nlm.nih.gov/bioproject/1423469]. Source data are provided with this paper.

## References

[1] Moran, N. A. Symbiosis as an adaptive process and source of phenotypic complexity. *Proc. Natl. Acad. Sci. USA*. **104**, 8627–8633 (2007). 10.1073/pnas.0611659104 PMC1876439 17494762

[2] Kaltenpoth, M., Flórez, L. V., Vigneron, A., Dirksen, P. & Engl, T. Origin and function of beneficial bacterial symbioses in insects. *Nat. Rev. Microbiol.* **23**, 551–567 (2025). 10.1038/s41579-025-01164-z 40148601

[3] Munson, M. A. et al. Evidence for the establishment of aphid eubacterium endosymbiosis in an ancestor of four aphid families. *J. Bacteriol.* **173**, 6321–6324 (1991). 1917864 10.1128/jb.173.20.6321-6324.1991 PMC208962

[4] Clark, M. A., Moran, N. A. & Baumann, P. Sequence evolution in bacterial endosymbionts having extreme base compositions. *Mol. Biol. Evol.* **16**, 1586–1598 (1999). 10555290 10.1093/oxfordjournals.molbev.a026071

[5] Moran, N. A., Tran, P. & Gerardo, N. M. Symbiosis and insect diversification: an ancient symbiont of sap-feeding insects from the bacterial phylum *Bacteroidetes*. *Appl Environ. Microbiol.* **71**, 8802–8810 (2005). 16332876 10.1128/AEM.71.12.8802-8810.2005 PMC1317441

[6] Engl, T. et al. Ancient symbiosis confers desiccation resistance to stored grain pest beetles. *Mol. Ecol.* **27**, 2095–2108 (2018). 29117633 10.1111/mec.14418

[7] Moran, N. A., McLaughlin, H. J. & Sorek, R. Dynamics and time scale of ongoing genomic erosion in symbiotic bacteria. *Science*. **323**, 379–382 (2009). 19150844 10.1126/science.1167140

[8] Luan, J. B. et al. Metabolic coevolution in the bacterial symbiosis of whiteflies and related plant sap-feeding insects. *Genome Biol. Evol.* **7**, 2635–2647 (2015). 26377567 10.1093/gbe/evv170 PMC4607527

[9] Wierz, J. C., Gimmel, M. L., Huthmacher, S., Engl, T. & Kaltenpoth, M. Evolutionary history of tyrosine-supplementing endosymbionts in pollen-feeding beetles. *ISME J.* **18**, wrae080 (2024). 10.1093/ismejo/wrae080 PMC11191362 38861456

[10] Kirsch, R. et al. Symbiosis and horizontal gene transfer promote herbivory in the megadiverse leaf beetles. *Curr. Biol.* **35**, 640–654 (2025). 39826554 10.1016/j.cub.2024.12.028

[11] Koga, R., Bennett, G. M., Cryan, J. R. & Moran, N. A. Evolutionary replacement of obligate symbionts in an ancient and diverse insect lineage. *Environ. Microbiol.* **15**, 2073–2081 (2013). 23574391 10.1111/1462-2920.12121

[12] Bennett, G. M. & Moran, N. A. Heritable symbiosis: The advantages and perils of an evolutionary rabbit hole. *Proc. Natl. Acad. Sci. USA*. **112**, 10169–10176 (2015). 25713367 10.1073/pnas.1421388112 PMC4547261

[13] Husnik, F. & McCutcheon, J. P. Repeated replacement of an intrabacterial symbiont in the tripartite nested mealybug symbiosis. *Proc. Natl. Acad. Sci. USA*. **113**, E5416–5424 (2016). 27573819 10.1073/pnas.1603910113 PMC5027413

[14] Sudakaran, S., Kost, C. & Kaltenpoth, M. Symbiont acquisition and replacement as a source of ecological innovation. *Trends Microbiol.* **25**, 375–390 (2017). 28336178 10.1016/j.tim.2017.02.014

[15] Cornwallis, C. K. et al. Symbioses shape feeding niches and diversification across insects. *Nat. Ecol. Evol.* **7**, 1022–1044 (2023). 37202501 10.1038/s41559-023-02058-0 PMC10333129

[16] Boscaro, V. et al. Symbiont replacement between bacteria of different classes reveals additional layers of complexity in the evolution of symbiosis in the ciliate *Euplotes*. *Protist*. **169**, 43–52 (2018). 29414319 10.1016/j.protis.2017.12.003

[17] Vannini, C., Sigona, C., Hahn, M., Petroni, G. & Fujishima, M. High degree of specificity in the association between symbiotic betaproteobacteria and the host *Euplotes* (Ciliophora, Euplotia). *Eur. J. Protistol.* **59**, 124–132 (2017). 28521174 10.1016/j.ejop.2017.04.003

[18] Fujishima, M. & Heckmann, K. Intra- and interspecies transfer of endosymbionts in *Euplotes*. *J. Exp. Zool.* **230**, 339–345 (1984).

[19] Su, Y. et al. Rational engineering of a synthetic insect-bacterial mutualism. *Curr. Biol.* **32**, 3925–3938 (2022). 35963240 10.1016/j.cub.2022.07.036 PMC10080585

[20] Grimaldi, D. & Engel, M. S. *Evolution of the Insects* (Cambridge University Press, 2005).

[21] Linz, D. M., Hu, A. W., Sitvarin, M. I. & Tomoyasu, Y. Functional value of elytra under various stresses in the red flour beetle, *Tribolium castaneum*. *Sci. Rep.* **6**, 34813 (2016). 27708390 10.1038/srep34813 PMC5052563

[22] Andersen, S. O. in *Comprehensive Molecular Insect Science* (ed L. I. Gilbert) 145–170 (Elsevier, 2005).

[23] Andersen, S. O. Insect cuticular sclerotization: a review. *Insect Biochem. Mol. Biol.* **40**, 166–178 (2010). 19932179 10.1016/j.ibmb.2009.10.007

[24] Duplais, C. et al. Gut bacteria are essential for normal cuticle development in herbivorous turtle ants. *Nat. Commun.* **12**, 676 (2021). 33514729 10.1038/s41467-021-21065-y PMC7846594

[25] Kanyile, S. N., Engl, T., Heddi, A. & Kaltenpoth, M. Endosymbiosis allows *Sitophilus oryzae* to persist in dry conditions. *Front. Microbiol.* **14**, 1199370 (2023). 37497544 10.3389/fmicb.2023.1199370 PMC10366622

[26] Kanyile, S. N., Engl, T. & Kaltenpoth, M. Nutritional symbionts enhance structural defence against predation and fungal infection in a grain pest beetle. *J. Exp. Biol.* **225**, jeb243593 (2022). 10.1242/jeb.243593 PMC8778805 34854911

[27] Chari, A. et al. Phenotypic characterization of *Sodalis praecaptivus* sp. nov., a close non-insect-associated member of the *Sodalis*-allied lineage of insect endosymbionts. *Int. J. Syst. Evol. Microbiol.* **65**, 1400–1405 (2015). 25782768 10.1099/ijs.0.000091 PMC4635462

[28] Koch, A. Die Symbiose von *Oryzaephilus surinamensis* L. (Cucujidae, Coleoptera). *Z. für. Morphologie und Ökologie der Tiere*. **23**, 389–424 (1931).

[29] Kiefer, J. S. T. et al. Inhibition of a nutritional endosymbiont by glyphosate abolishes mutualistic benefit on cuticle synthesis in *Oryzaephilus surinamensis*. *Commun. Biol.* **4**, 554 (2021). 10.1038/s42003-021-02057-6 PMC8113238 33976379

[30] Ewald, P. W. Transmission modes and evolution of the parasitism-mutualism continuum. *Ann. N. Y. Acad. Sci.* **503**, 295–306 (1987). 3304078 10.1111/j.1749-6632.1987.tb40616.x

[31] Frank, S. A. Models of symbiosis. *Am. Nat.* **150**, S80–99 (1997). 18811314 10.1086/286051

[32] Matsuura, Y. et al. Recurrent symbiont recruitment from fungal parasites in cicadas. *Proc. Natl. Acad. Sci. USA*. **115**, E5970–E5979 (2018). 29891654 10.1073/pnas.1803245115 PMC6042066

[33] Chong, R. A. & Moran, N. A. Evolutionary loss and replacement of *Buchnera*, the obligate endosymbiont of aphids. *ISME J.* **12**, 898–908 (2018). 29362506 10.1038/s41396-017-0024-6 PMC5864228

[34] Lefevre, C. et al. Endosymbiont phylogenesis in the Dryophthoridae weevils: evidence for bacterial replacement. *Mol. Biol. Evol.* **21**, 965–973 (2004). 14739242 10.1093/molbev/msh063

[35] Hurst, G. D., Anbutsu, H., Kutsukake, M. & Fukatsu, T. Hidden from the host: *Spiroplasma* bacteria infecting *Drosophila* do not cause an immune response, but are suppressed by ectopic immune activation. *Insect Mol. Biol.* **12**, 93–97 (2003). 12542640 10.1046/j.1365-2583.2003.00380.x

[36] Anbutsu, H. & Fukatsu, T. Evasion, suppression and tolerance of *Drosophila* innate immunity by a male-killing *Spiroplasma* endosymbiont. *Insect Mol. Biol.* **19**, 481–488 (2010). 20456506 10.1111/j.1365-2583.2010.01008.x

[37] Zhang, Q., Wei, X., Fang, W., Huang, X. & Zhang, X. The secretory protein COA1 enables *Metarhizium robertsii* to evade insect immune recognition during cuticle penetration. *Commun. Biol.* **7**, 1220 (2024). 39349686 10.1038/s42003-024-06827-w PMC11442803

[38] Hu, Y. & Aksoy, S. An antimicrobial peptide with trypanocidal activity characterized from *Glossina morsitans morsitans*. *Insect Biochem. Mol. Biol.* **35**, 105–115 (2005). 15681221 10.1016/j.ibmb.2004.10.007

[39] Pontes, M. H., Smith, K. L., De Vooght, L., Van Den Abbeele, J. & Dale, C. Attenuation of the sensing capabilities of PhoQ in transition to obligate insect-bacterial association. *PLoS Genet.* **7**, e1002349 (2011). 22072980 10.1371/journal.pgen.1002349 PMC3207850

[40] Clayton, A. L., Enomoto, S., Su, Y. & Dale, C. The regulation of antimicrobial peptide resistance in the transition to insect symbiosis. *Mol. Microbiol.* **103**, 958–972 (2017). 27987256 10.1111/mmi.13598 PMC5344769

[41] Anselme, C., Vallier, A., Balmand, S., Fauvarque, M. O. & Heddi, A. Host PGRP gene expression and bacterial release in endosymbiosis of the weevil *Sitophilus zeamais*. *Appl. Environ. Microbiol.* **72**, 6766–6772 (2006). 17021229 10.1128/AEM.00942-06 PMC1610295

[42] Maire, J. et al. Weevil pgrp-lb prevents endosymbiont TCT dissemination and chronic host systemic immune activation. *Proc. Natl. Acad. Sci. USA*. **116**, 5623–5632 (2019). 30819893 10.1073/pnas.1821806116 PMC6431197

[43] Bright, M. & Bulgheresi, S. A complex journey: transmission of microbial symbionts. *Nat. Rev. Microbiol.* **8**, 218–230 (2010). 20157340 10.1038/nrmicro2262 PMC2967712

[44] Rio, R. V. et al. Insight into the transmission biology and species-specific functional capabilities of tsetse (Diptera: Glossinidae) obligate symbiont Wigglesworthia. *mBio*. **3**, e00240–11 (2012). 10.1128/mBio.00240-11 PMC3280448 22334516

[45] Santos-Garcia, D. et al. The genome of *Cardinium* cBtQ1 provides insights into genome reduction, symbiont motility, and its settlement in *Bemisia tabaci*. *Genome Biol. Evol.* **6**, 1013–1030 (2014). 24723729 10.1093/gbe/evu077 PMC4007549

[46] Lee, J. B. et al. Bacterial cell motility of *Burkholderia* gut symbiont is required to colonize the insect gut. *FEBS Lett.* **589**, 2784–2790 (2015). 26318755 10.1016/j.febslet.2015.08.022

[47] Dale, C., Young, S. A., Haydon, D. T. & Welburn, S. C. The insect endosymbiont *Sodalis glossinidius* utilizes a ype III secretion system for cell invasion. *PNAS*. **98**, 1883–1888 (2000). 10.1073/pnas.021450998 PMC29351 11172045

[48] Deng, W. et al. Assembly, structure, function and regulation of type III secretion systems. *Nat. Rev. Microbiol.* **15**, 323–337 (2017). 28392566 10.1038/nrmicro.2017.20

[49] Teulet, A., Camuel, A., Perret, X. & Giraud, E. The versatile roles of type III secretion systems in rhizobium-legume symbioses. *Annu. Rev. Microbiol.* **76**, 45–65 (2022). 35395168 10.1146/annurev-micro-041020-032624

[50] Su, Y., Lin, H. C. & Dale, C. Protocol to establish a genetically tractable synthetic symbiosis between *Sodalis praecaptivus* and grain weevils by insect egg microinjection. *STAR Protoc.* **4**, 102156 (2023). 36917608 10.1016/j.xpro.2023.102156 PMC10025269

[51] Ankrah, N. Y. D., Luan, J. & Douglas, A. E. Cooperative metabolism in a three-partner insect-bacterial symbiosis revealed by metabolic modeling. *J. Bacteriol.* **199**, e00872–16 (2017). 10.1128/JB.00872-16 PMC5512215 28348026

[52] Goto, S., Anbutsu, H. & Fukatsu, T. Asymmetrical interactions between *Wolbachia* and *Spiroplasma* endosymbionts coexisting in the same insect host. *Appl. Environ. Microbiol.* **72**, 4805–4810 (2006). 16820474 10.1128/AEM.00416-06 PMC1489378

[53] Koga, R., Tsuchida, T. & Fukatsu, T. Changing partners in an obligate symbiosis: a facultative endosymbiont can compensate for loss of the essential endosymbiont *Buchnera* in an aphid. *Proc. Biol. Sci.* **270**, 2543–2550 (2003). 14728775 10.1098/rspb.2003.2537 PMC1691542

[54] Huang, Y., Feng, Z. F., Li, F. & Hou, Y. M. Host-encoded aminotransferase import into the endosymbiotic bacteria *Nardonella*of Red Palm Weevil. *Insects*. **15**, 35 (2024). 10.3390/insects15010035 PMC10816905 38249041

[55] Halary, S., Duperron, S. & Boudier, T. Direct image-based correlative microscopy technique for coupling identification and structural investigation of bacterial symbionts associated with metazoans. *Appl. Environ. Microbiol.* **77**, 4172–4179 (2011). 21515722 10.1128/AEM.02461-10 PMC3131643

[56] Vigneron, A. et al. Insects recycle endosymbionts when the benefit is over. *Curr. Biol.* **24**, 2267–2273 (2014). 25242028 10.1016/j.cub.2014.07.065

[57] Carvalho, A. S. P. et al. Symbionts with eroded genomes adjust gene expression according to host life-stage and environment. *EMBO Rep.* **26**, 4656–4674 (2025). 40781167 10.1038/s44319-025-00525-2 PMC12508126

[58] Cabeen, M. T. & Jacobs-Wagner, C. Bacterial cell shape. *Nat. Rev. Microbiol.* **3**, 601–610 (2005). 16012516 10.1038/nrmicro1205

[59] McCutcheon, J. P., Boyd, B. M. & Dale, C. The life of an insect endosymbiont from the cradle to the grave. *Curr. Biol.* **29**, R485–R495 (2019). 31163163 10.1016/j.cub.2019.03.032

[60] Florez, L. V., Biedermann, P. H., Engl, T. & Kaltenpoth, M. Defensive symbioses of animals with prokaryotic and eukaryotic microorganisms. *Nat. Prod. Rep.* **32**, 904–936 (2015). 25891201 10.1039/c5np00010f

[61] Engl, T. et al. Evolutionary stability of antibiotic protection in a defensive symbiosis. *Proc. Natl. Acad. Sci. USA*. **115**, E2020–E2029 (2018). 29444867 10.1073/pnas.1719797115 PMC5834716

[62] Oliver, K. M., Degnan, P. H., Hunter, M. S. & Moran, N. A. Bacteriophages encode factors required for protection in a symbiotic mutualism. *Science*. **325**, 992–994 (2009). 19696350 10.1126/science.1174463 PMC5473335

[63] Scarborough, C. L., Ferrari, J. & Godfray, H. C. J. Aphid protected from pathogen by endosymbiont. *Science*. **310**, 1781 (2005). 16357252 10.1126/science.1120180

[64] Pande, S. et al. Fitness and stability of obligate cross-feeding interactions that emerge upon gene loss in bacteria. *ISME J.* **8**, 953–962 (2014). 24285359 10.1038/ismej.2013.211 PMC3996690

[65] Scarinci, G. et al. Enhanced metabolic entanglement emerges during the evolution of an interkingdom microbial community. *Nat. Commun.* **15**, 7238 (2024). 39174531 10.1038/s41467-024-51702-1 PMC11341674

[66] Koga, R. et al. Single mutation makes *Escherichia coli* an insect mutualist. *Nat. Microbiol.* **7**, 1141–1150 (2022). 35927448 10.1038/s41564-022-01179-9 PMC9352592

[67] Rogowska-van der Molen, M. A. et al. From eggs to guts: symbiotic association of *Sodalis nezarae sp. nov*. with the southern green shield bug *Nezara viridula*. *FEMS Microbiol. Ecol.* **101**, fiaf017 (2025). 10.1093/femsec/fiaf017 PMC11879575 39938947

[68] Santos-Garcia, D., Silva, F. J., Morin, S., Dettner, K. & Kuechler, S. M. The All-Rounder *Sodalis*: a new bacteriome-associated endosymbiont of the lygaeoid bug *Henestaris halophilus* (Heteroptera: Henestarinae) and a critical examination of its evolution. *Genome Biol. Evol.* **9**, 2893–2910 (2017). 29036401 10.1093/gbe/evx202 PMC5737371

[69] Boyd, B. M. et al. Two bacterial genera, *Sodalis* and *Rickettsia*, associated with the seal louse *Proechinophthirus fluctus* (Phthiraptera: Anoplura). *Appl. Environ. Microbiol.* **82**, 3185–3197 (2016). 10.1128/AEM.00282-16 PMC4959230 26994086

[70] Fukatsu, T. et al. Bacterial endosymbiont of the slender pigeon louse, *Columbicola columbae*, allied to endosymbionts of grain weevils and tsetse flies. *Appl Environ. Microbiol.* **73**, 6660–6668 (2007). 17766458 10.1128/AEM.01131-07 PMC2075037

[71] Dale, C. & Maudlin, I. *Sodalis* gen. nov. and *Sodalis glossinidius* sp. nov., a microaerophilic secondary endosymbiont of the tsetse fly *Glossina morsitans morsitans*. *Int. J. Syst. Evolut. Microbiol.* **49**, 267–275 (1999). 10.1099/00207713-49-1-267 10028272

[72] Engl, T., Schmidt, T. H. P., Kanyile, S. N. & Klebsch, D. Metabolic cost of a nutritional symbiont manifests in delayed reproduction in a grain pest beetle. *Insects*. **11**, 717 (2020). 10.3390/insects11100717 PMC7589553 33092035

[73] Medina Munoz, M., Spencer, N., Enomoto, S., Dale, C. & Rio, R. V. M. Quorum sensing sets the stage for the establishment and vertical transmission of *Sodalis praecaptivus* in tsetse flies. *PLoS Genet.* **16**, e1008992 (2020). 32797092 10.1371/journal.pgen.1008992 PMC7449468

[74] Tanahashi, M. & Fukatsu, T. Natsumushi: image measuring software for entomological studies. *Entomol. Sci.* **21**, 347–360 (2018).

[75] Janke, R. S., Moog, S., Weiss, B., Kaltenpoth, M. & Florez, L. V. Morphological adaptation for ectosymbiont maintenance and transmission during metamorphosis in *Lagria* beetles. *Front. Physiol.* **13**, 979200 (2022). 36111144 10.3389/fphys.2022.979200 PMC9468232

[76] Comet Technologies Canada Inc., Montreal Canada, Dragonfly V2022.2.

[77] Weiss, B. *Techniques of Insect Histology. A Guideline for the Preparation of Insects for Light Microscopic Analysis* (Shaker Verlag, 2023).

[78] Shalom, S. R., Weiss, B., Lalzar, M., Kaltenpoth, M. & Chiel, E. Abundance and localization of symbiotic bacterial communities in the fly parasitoid *Spalangia cameroni*. *Appl. Environ. Microbiol.* **88**, e02549–02521 (2022). 35420439 10.1128/aem.02549-21 PMC9088259

[79] Amann, R. I. et al. Combination of 16S rRNA-targeted oligonucleotide probes with flow cytometry for analyzing mixed microbial populations. *Appl. Environ. Microbiol.* **56**, 1919–1925 (1990). 2200342 10.1128/aem.56.6.1919-1925.1990 PMC184531

[80] FelixKrueger/TrimGalore: v0.6.10 - add default decompression path (0.6.10) (Zenodo, 2023).

[81] Kopylova, E., Noe, L. & Touzet, H. SortMeRNA: fast and accurate filtering of ribosomal RNAs in metatranscriptomic data. *Bioinformatics*. **28**, 3211–3217 (2012). 23071270 10.1093/bioinformatics/bts611

[82] Ewels, P. A. et al. The nf-core framework for community-curated bioinformatics pipelines. *Nat. Biotechnol.* **38**, 276–278 (2020). 32055031 10.1038/s41587-020-0439-x

[83] Schwengers, O. et al. Bakta: rapid and standardized annotation of bacterial genomes via alignment-free sequence identification. *Microbial Genomics*. **7**, 000685 (2021). 10.1099/mgen.0.000685 PMC8743544 34739369

[84] Kanehisa, M. G. S. KEGG: Kyoto Encyclopedia of Genes and Genomes. *Nucleic Acids Res.* **28**, 27–30 (2000). 10592173 10.1093/nar/28.1.27 PMC102409

[85] Kanehisa, M., Sato, Y. & Morishima, K. BlastKOALA and GhostKOALA: KEGG tools for functional characterization of genome and metagenome sequences. *J. Mol. Biol.* **428**, 726–731 (2016). 26585406 10.1016/j.jmb.2015.11.006

[86] *R: A Language and Environment for Statistical Computing* (R Foundation for Statistical Computing, Vienna, Austria, 2024).

[87] Langmead, B. & Salzberg, S. L. Fast gapped-read alignment with Bowtie 2. *Nat. Methods*. **9**, 357–359 (2012). 22388286 10.1038/nmeth.1923 PMC3322381

[88] Danecek, P. et al. Twelve years of SAMtools and BCFtools. *Gigascience*. **10**, giab008 (2021). 10.1093/gigascience/giab008 PMC7931819 33590861

[89] Chung, M. A. et al. FADU: a quantification tool for prokaryotic transcriptomic analyses. *mSystems*. **6**, e00917–00920 (2021). 33436511 10.1128/mSystems.00917-20 PMC7901478

[90] Wick, R. R., Judd, L. M. & Holt, K. E. Performance of neural network basecalling tools for Oxford Nanopore sequencing. *Genome Biol.* **20**, 129 (2019). 31234903 10.1186/s13059-019-1727-y PMC6591954

[91] Kolmogorov, M. et al. metaFlye: scalable long-read metagenome assembly using repeat graphs. *Nat. Methods*. **17**, 1103–1110 (2020). 33020656 10.1038/s41592-020-00971-x PMC10699202

[92] Vaser, R., Sovic, I., Nagarajan, N. & Sikic, M. Fast and accurate de novo genome assembly from long uncorrected reads. *Genome Res.* **27**, 737–746 (2017). 28100585 10.1101/gr.214270.116 PMC5411768

[93] Warren, R. L. et al. ntEdit: scalable genome sequence polishing. *Bioinformatics*. **35**, 4430–4432 (2019). 31095290 10.1093/bioinformatics/btz400 PMC6821332

[94] Laetsch, D. R. & Blaxter, M. L. BlobTools: interrogation of genome assemblies. *F1000Research*. **6**, 1287 (2017).

[95] Manni, M., Berkeley, M. R., Seppey, M., Simao, F. A. & Zdobnov, E. M. BUSCO update: novel and streamlined workflows along with broader and deeper phylogenetic coverage for scoring of eukaryotic, prokaryotic, and viral genomes. *Mol. Biol. Evol.* **38**, 4647–4654 (2021). 34320186 10.1093/molbev/msab199 PMC8476166

[96] Stanke, M., Diekhans, M., Baertsch, R. & Haussler, D. Using native and syntenically mapped cDNA alignments to improve de novo gene finding. *Bioinformatics*. **24**, 637–644 (2008). 18218656 10.1093/bioinformatics/btn013

[97] Stanke, M., Schoffmann, O., Morgenstern, B. & Waack, S. Gene prediction in eukaryotes with a generalized hidden Markov model that uses hints from external sources. *BMC Bioinform.* **7**, 62 (2006). 10.1186/1471-2105-7-62 PMC1409804 16469098

[98] Gabriel, L., Hoff, K. J., Bruna, T., Borodovsky, M. & Stanke, M. TSEBRA: transcript selector for BRAKER. *BMC Bioinform.* **22**, 566 (2021). 10.1186/s12859-021-04482-0 PMC8620231 34823473

[99] Bruna, T., Lomsadze, A. & Borodovsky, M. GeneMark-EP+: eukaryotic gene prediction with self-training in the space of genes and proteins. *NAR Genom. Bioinform.* **2**, lqaa026 (2020). 32440658 10.1093/nargab/lqaa026 PMC7222226

[100] Buchfink, B., Xie, C. & Huson, D. H. Fast and sensitive protein alignment using DIAMOND. *Nat. Methods*. **12**, 59–60 (2015). 25402007 10.1038/nmeth.3176

[101] Lomsadze, A., Ter-Hovhannisyan, V., Chernoff, Y. O. & Borodovsky, M. Gene identification in novel eukaryotic genomes by self-training algorithm. *Nucleic Acids Res.* **33**, 6494–6506 (2005). 16314312 10.1093/nar/gki937 PMC1298918

[102] Iwata, H. & Gotoh, O. Benchmarking spliced alignment programs including Spaln2, an extended version of Spaln that incorporates additional species-specific features. *Nucleic Acids Res.* **40**, e161–e161 (2012). 22848105 10.1093/nar/gks708 PMC3488211

[103] Gotoh, O., Morita, M. & Nelson, D. R. Assessment and refinement of eukaryotic gene structure prediction with gene-structure-aware multiple protein sequence alignment. *BMC Bioinform.* **15**, 189 (2014). 10.1186/1471-2105-15-189 PMC4065584 24927652

[104] Gabriel, L. et al. BRAKER3: fully automated genome annotation using RNA-seq and protein evidence with GeneMark-ETP, AUGUSTUS, and TSEBRA. *Genome Res.* **34**, 769–777 (2024). 38866550 10.1101/gr.278090.123 PMC11216308

[105] NBISweden/AGAT: AGAT v. v1.5.1 (Zenodo, 2025).

[106] Tarailo-Graovac, M. & Chen, N. Using RepeatMasker to identify repetitive elements in genomic sequences. *Curr. Protoc. Bioinform.* **4**, 4 10 11–14 10 14 (2009). 10.1002/0471250953.bi0410s25 19274634

[107] Flynn, J. M. et al. RepeatModeler2 for automated genomic discovery of transposable element families. *Proc. Natl. Acad. Sci. USA*. **117**, 9451–9457 (2020). 32300014 10.1073/pnas.1921046117 PMC7196820

[108] Pertea, G. & Pertea, M. GFF Utilities: GffRead and GffCompare. *F1000Res*. **9**, 304 (2020). 10.12688/f1000research.23297.1 PMC7222033 32489650

[109] Huerta-Cepas, J. et al. eggNOG 5.0: a hierarchical, functionally and phylogenetically annotated orthology resource based on 5090 organisms and 2502 viruses. *Nucleic Acids Res.* **47**, D309–D314 (2019). 30418610 10.1093/nar/gky1085 PMC6324079

[110] Jones, P. et al. InterProScan 5: genome-scale protein function classification. *Bioinformatics*. **30**, 1236–1240 (2014). 24451626 10.1093/bioinformatics/btu031 PMC3998142

[111] Chen, Y., Chen, L., Lun, A. T. L., Baldoni, P. L. & Smyth, G. K. edgeR v4: powerful differential analysis of sequencing data with expanded functionality and improved support for small counts and larger datasets. *Nucleic Acids Res.* **53**, gkaf018 (2025). 10.1093/nar/gkaf018 PMC11754124 39844453

[112] Love, M. I., Huber, W. & Anders, S. Moderated estimation of fold change and dispersion for RNA-seq data with DESeq2. *Genome Biol.* **15**, 550 (2014). 25516281 10.1186/s13059-014-0550-8 PMC4302049

[113] Ripley, B. D. Spatial statistics in R. *R. N.* **1**, 14–15 (2001).

[114] Zhu, A., Ibrahim, J. G. & Love, M. I. Heavy-tailed prior distributions for sequence count data: removing the noise and preserving large differences. *Bioinformatics*. **35**, 2084–2092 (2019). 30395178 10.1093/bioinformatics/bty895 PMC6581436

[115] Therneau, T. M. *coxme: mixed effects Cox models* https://CRAN.R-project.org/package=coxme (The Comprehensive R Archive Network, 2024).

[116] Fox, J. & Weisberg, S. *An R Companion to Applied Regression* (Sage, Thousand Oaks CA, 2019).

[117] Zeileis, A., Kleiber, C. & Jackman, S. Regression models for count data in R. *J. Stat. Softw. are*. **27**, 1–25 (2008).

[118] Wickham, H. *ggplot2: Elegant Graphics for Data Analysis* (Springer-Verlag New York, 2016).

