# Supplementary material for: Experimental Sodalis infection eliminates ancient insect symbiont
Source: Nat Commun. 2026 Mar 31;17:3153. doi: 10.1038/s41467-026-71143-2 (PMC13043703; doi:10.1038/s41467-026-71143-2)
Supplement: Supplementary file 1 — Supplementary Information [file 41467_2026_71143_MOESM1_ESM.pdf]

Supplementary Information

Experimental *Sodalis* infection eliminates ancient insect symbiont

Ronja Krüsemer[1], Ana S. P. Carvalho[1], Jean Keller[1,2], Heiko Vogel[1], Colin Dale[3], Tobias Engl[1], Martin Kaltenpoth[1*]

[1] Department of Insect Symbiosis, Max Planck Institute for Chemical Ecology, Jena, Germany

[2] Laboratoire de Recherche en Sciences Végétales, Université de Toulouse, CNRS, UPS, Toulouse INP, Castanet-Tolosan, France

[3] School of Biological Sciences, University of Utah, Salt Lake City, UT 84112, USA

[*] Corresponding author, kaltenpoth@ice.mpg.de

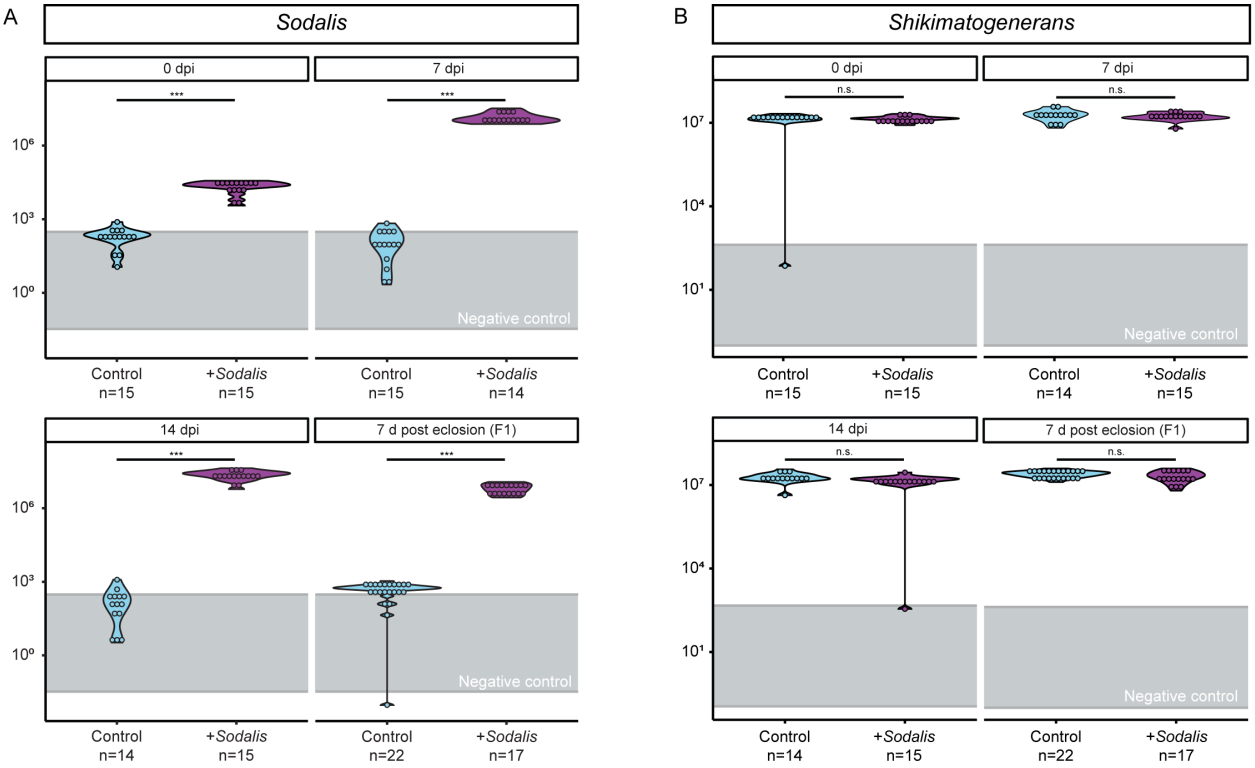

Supplementary Figure 1: Copy numbers of *Sodalis* and *Shikimatogenerans* in parental and first offspring generation of *O. surinamensis*. a *tam* gene copy number of *Sodalis* in adult *O. surinamensis* females 0, 7, and 14 days post injection (dpi) as well as adult beetle offspring 7 days post eclosion. **b** 16S copy number of *Shikimatogenerans* in adult *O. surinamensis* females 0, 7, and 14 days post injection (dpi) as well as adult beetle offspring 7 days post eclosion. Grey area indicates values measured in negative controls. Pairwise comparisons between the two treatment groups were made using two-sided Wilcoxon rank sum test or t-tests. Significant differences between *Sodalis*-infected and control beetles are indicated by asterisks (*** equals P<0.001). Source Data are provided as a Source Data file.

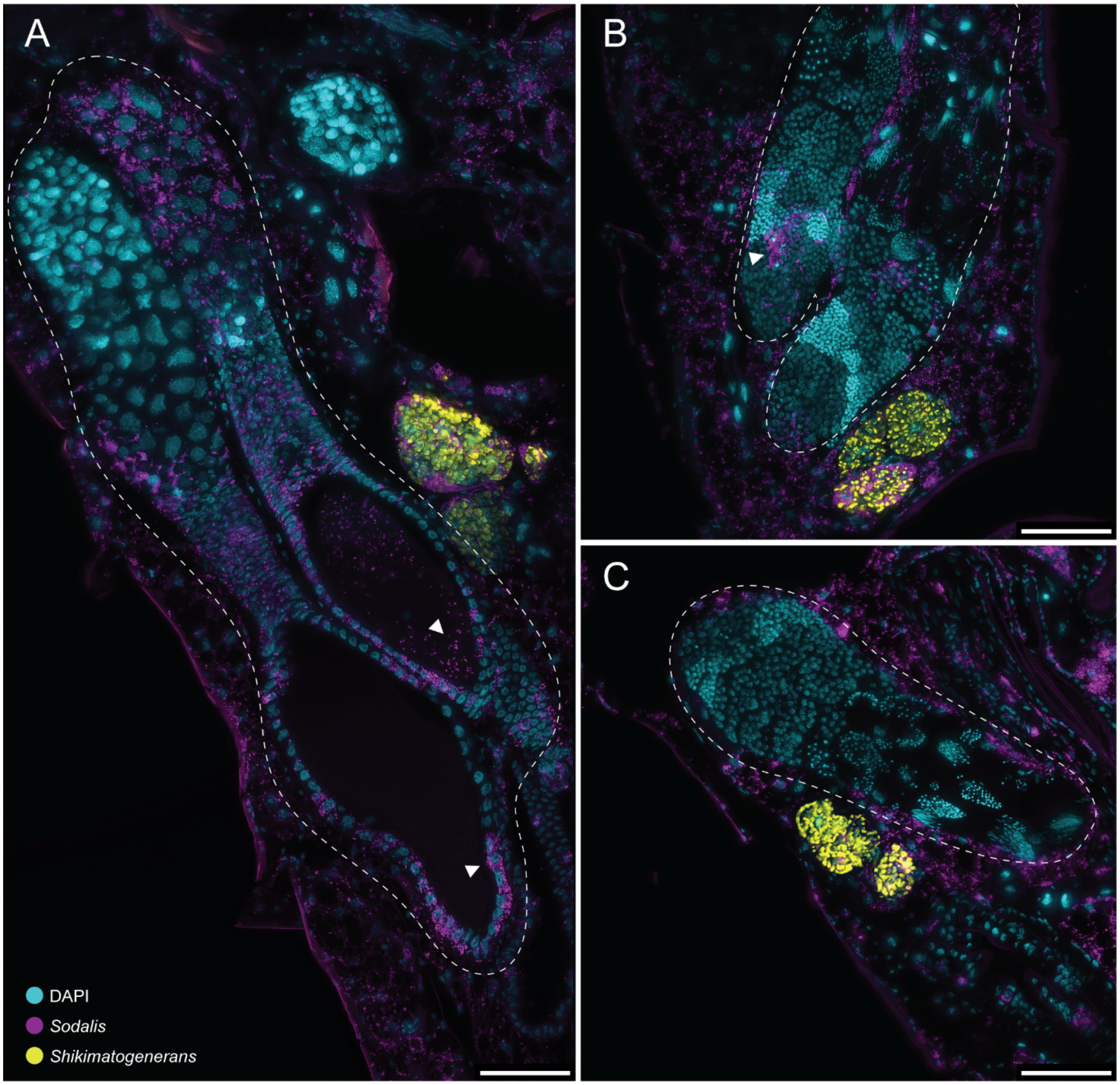

Supplementary Figure 2: FISH micrographs of *Sodalis* in beetle reproductive organs. a *O. surinamensis* ovary with *Sodalis* in developing eggs, indicating transovarial vertical transmission. **b,c** *O. surinamensis* testes with (b) and without (c) *Sodalis.* White arrows highlight accumulations of *Sodalis.* DNA was stained with DAPI (cyan) while the probes OsurSym16S-Cy5 and Sod-FISH-Cy3 were used to label *Shikimatogenerans* (yellow) and *Sodalis* (magenta), respectively. Observations were replicated independently with similar results (*Sodalis* in eggs: n=4, *Sodalis* in male testes: n=2, no *Sodalis* infection of male testes: n=2). Scale bars represent 50 µm.

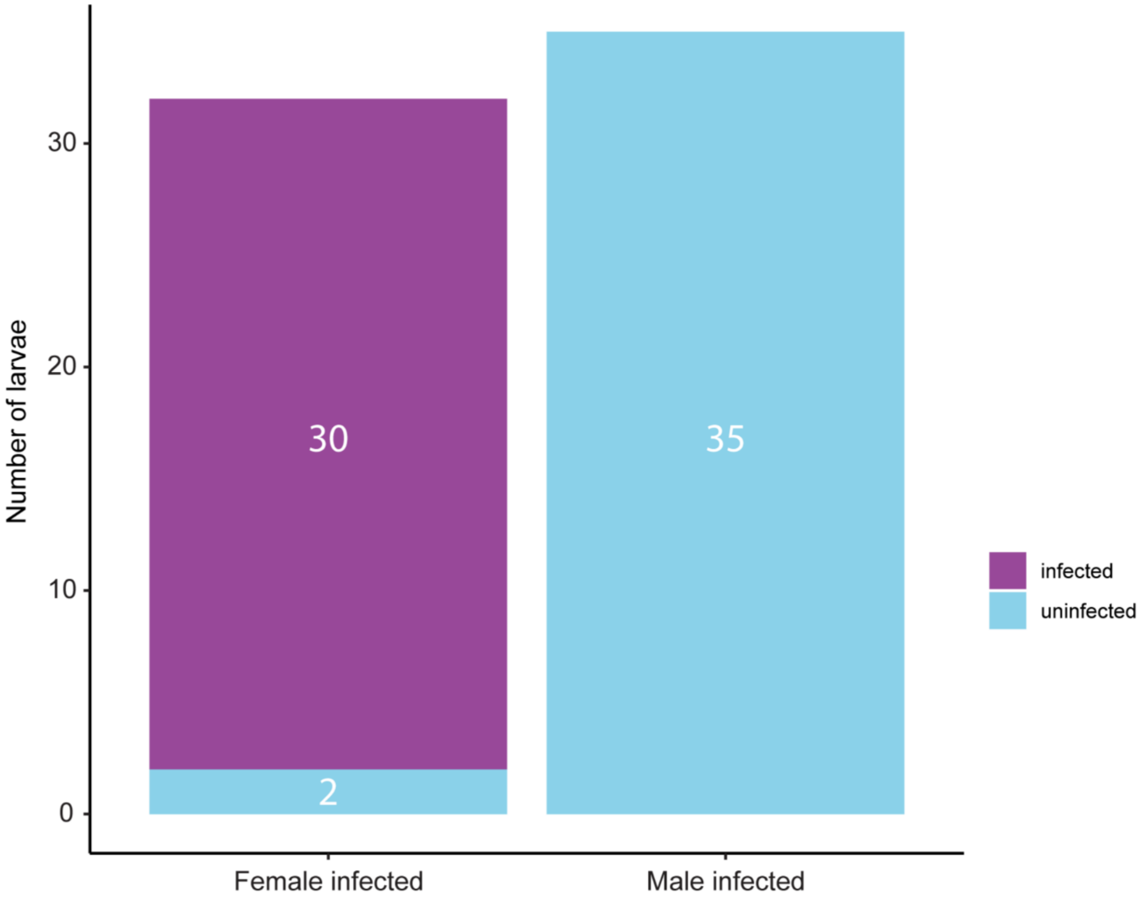

Supplementary Figure 3: **Efficiency of *Sodalis* transmission from infected parents to their offspring**. Stacked barplot displaying the total number of larvae produced by beetle mating pairs in which either the female (n=31 mating pairs of which 8 produced offspring) or male (n=19 mating pairs of which 13 produced offspring) was infected with *Sodalis*. Light blue bars indicate the uninfected and purple bars the infected fraction of F1 offspring. *Sodalis* was vertically transmitted from mother to offspring with an efficiency of 93.5%, while male beetles failed to transmit *Sodalis*. Source Data are provided as a Source Data file.

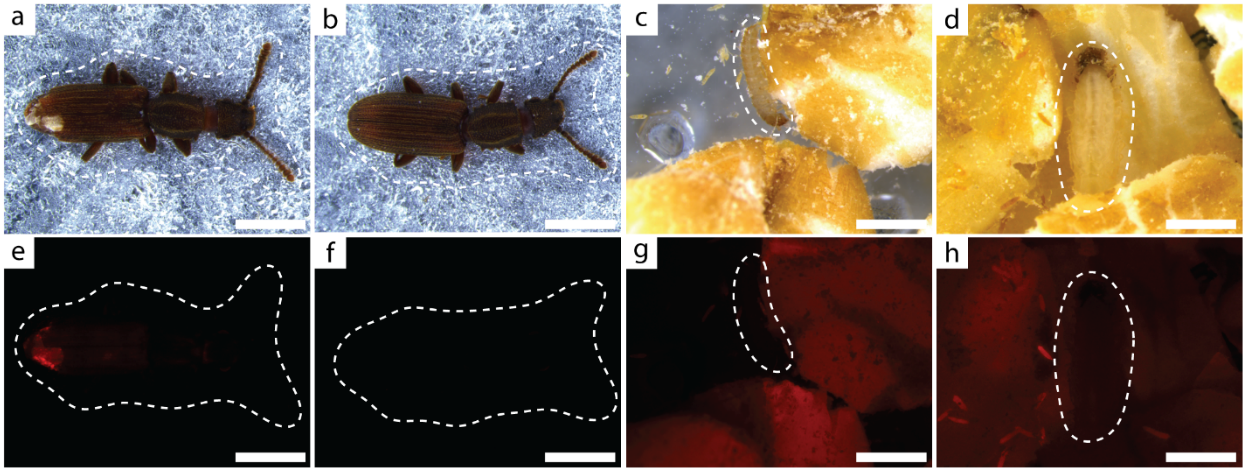

Supplementary Figure 4: *Sodalis* is not transmitted paternally. a,e Brightfield and fluorescence microscopic images of *O. surinamensis* males injected with *Sodalis*. **b,f** A female beetle that mated with an infected male did not acquire *Sodalis* horizontally during mating. **c-d, g-h** First generation offspring from infected males and uninfected females. No presence of *Sodalis* was observed and supports the absence of paternal transmission. Observations were replicated independently with similar results (n=19 mating pairs, in which all males but no females exhibited fluorescence, n=35 larvae produced by 13 mating pairs of which none were infected with *Sodalis*). Scale bar equals 1 mm.

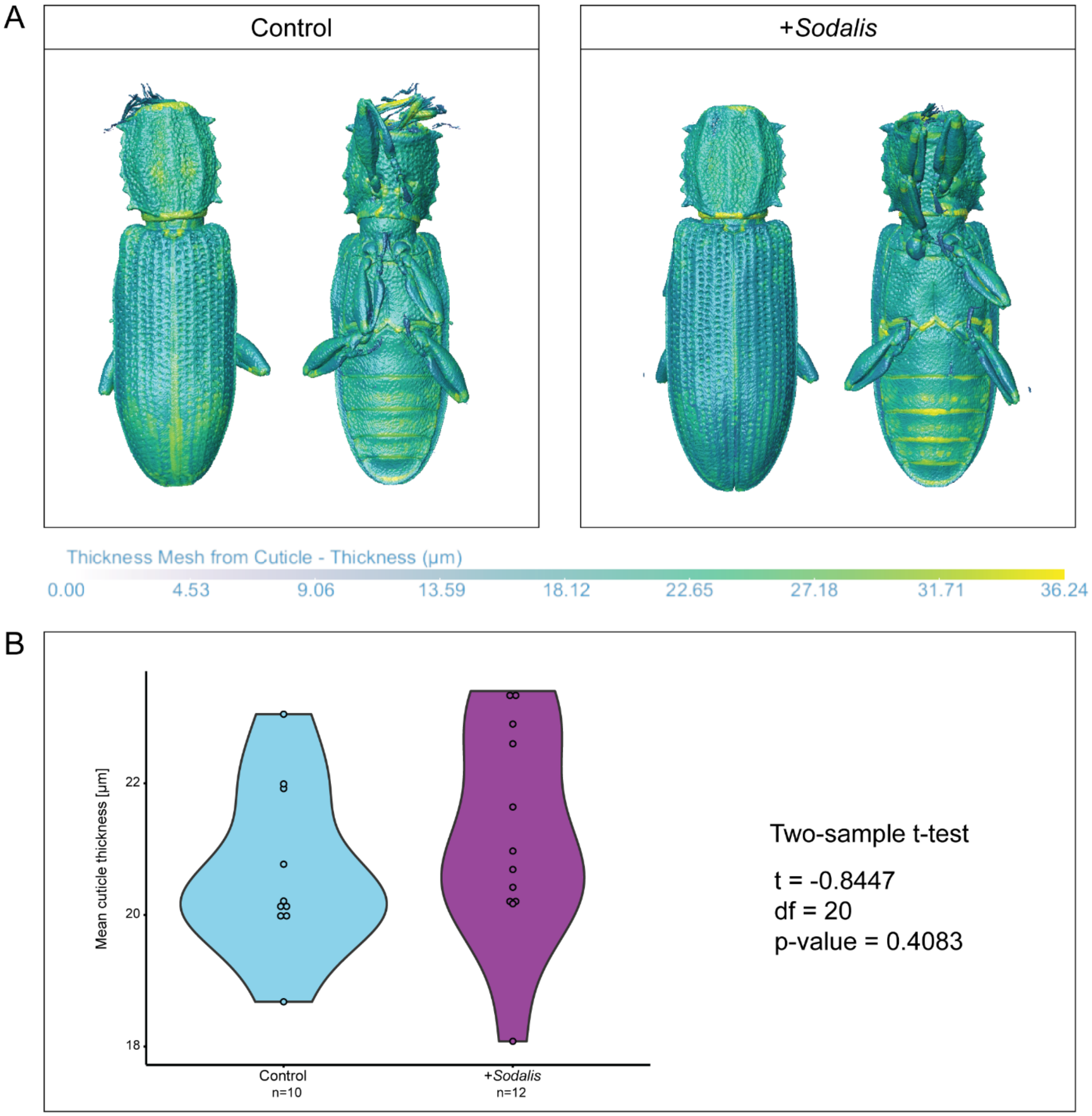

Supplementary Figure 5: Mean cuticle thickness based on 3D reconstructions of µCT datasets. a Cuticle thickness mesh applied to 3D reconstructions of *O. surinamensis* adults of the first offspring generation seven days after eclosion. **b** Mean cuticle thickness extracted from thickness meshes did not differ between the two treatments. Source Data are provided as a Source Data file.

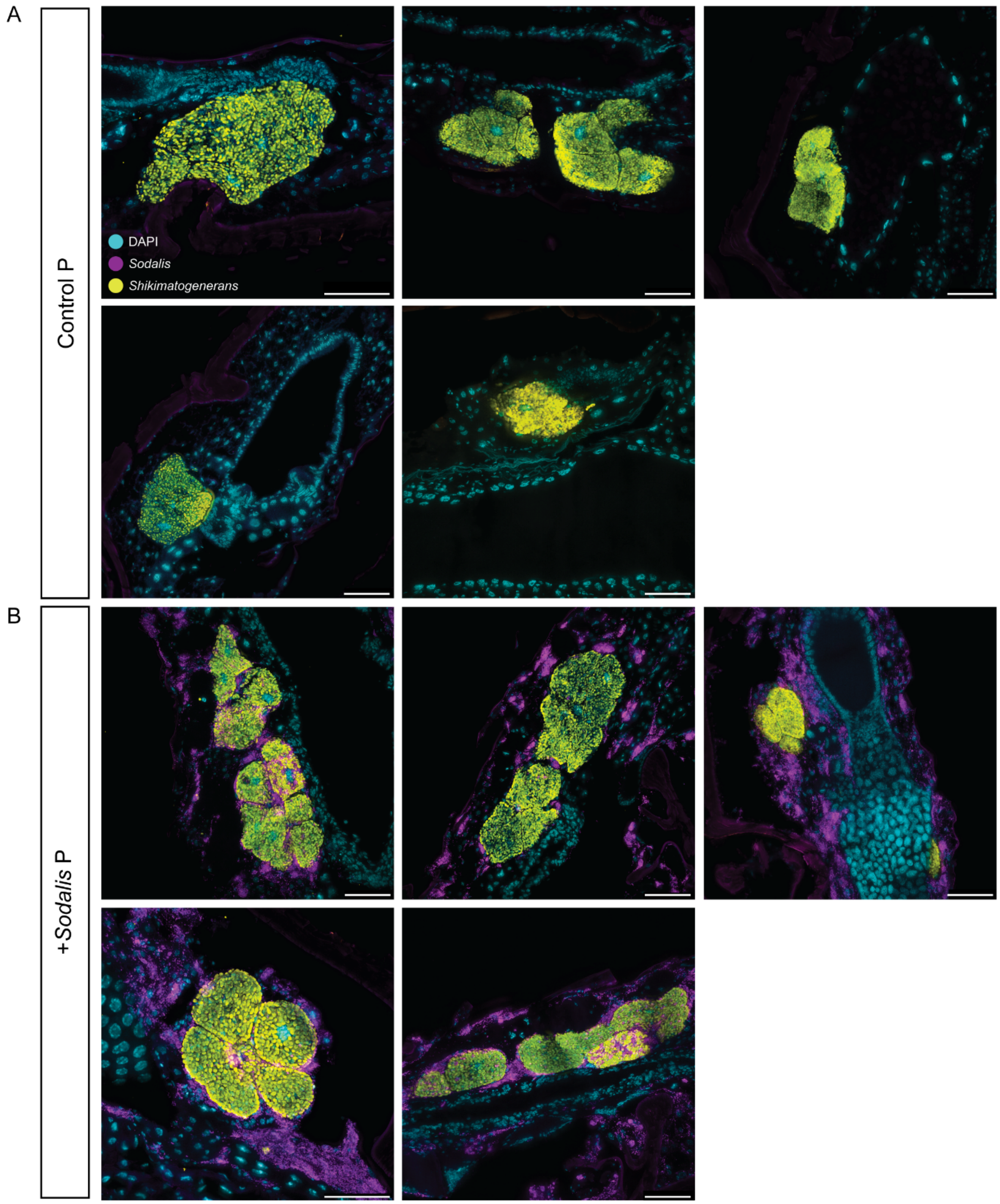

Supplementary Figure 6: FISH micrographs of control and *Sodalis*-injected *O. surinamensis* females. a Micrographs of beetles in the control treatment injected with 0.85% NaCl (w/v) (n=5) **b** Micrographs of beetles injected with *Sodalis* suspension demonstrating the presence of *Sodalis* across beetle tissues (n=5). DNA was stained with DAPI (cyan), while the probes OsurSym16S-Cy5 and Sod-FISH-Cy3 were used to label *Shikimatogenerans* (yellow) and *Sodalis* (magenta), respectively. Scale bars equal 50 µm.

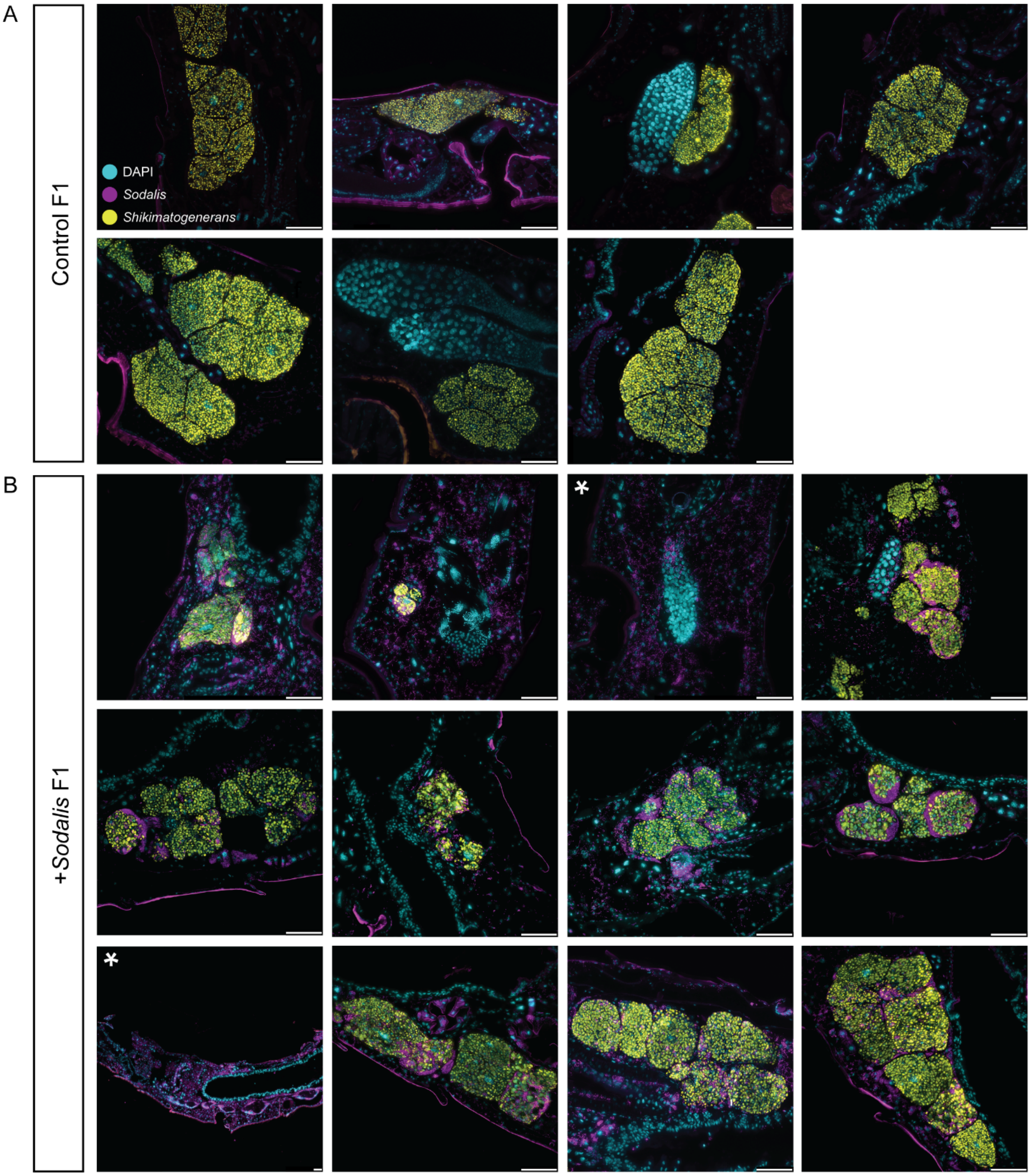

Supplementary Figure 7: FISH micrographs of first generation offspring of control and *Sodalis*-injected *O. surinamensis* females. a Micrographs of first generation offspring of control beetles injected with 0.85% NaCl (w/v) solution (n=7). **b** Micrographs of first generation offspring of beetles injected with *Sodalis* suspension (n=12). Asterisks indicate samples in which the ancient symbiont *Shikimatogenerans* was lost. DNA was stained with DAPI (cyan), while the probes OsurSym16S-Cy5 and Sod-FISH-Cy3 were used to label *Shikimatogenerans* (yellow) and *Sodalis* (magenta), respectively. Scale bars equal 50 µm.

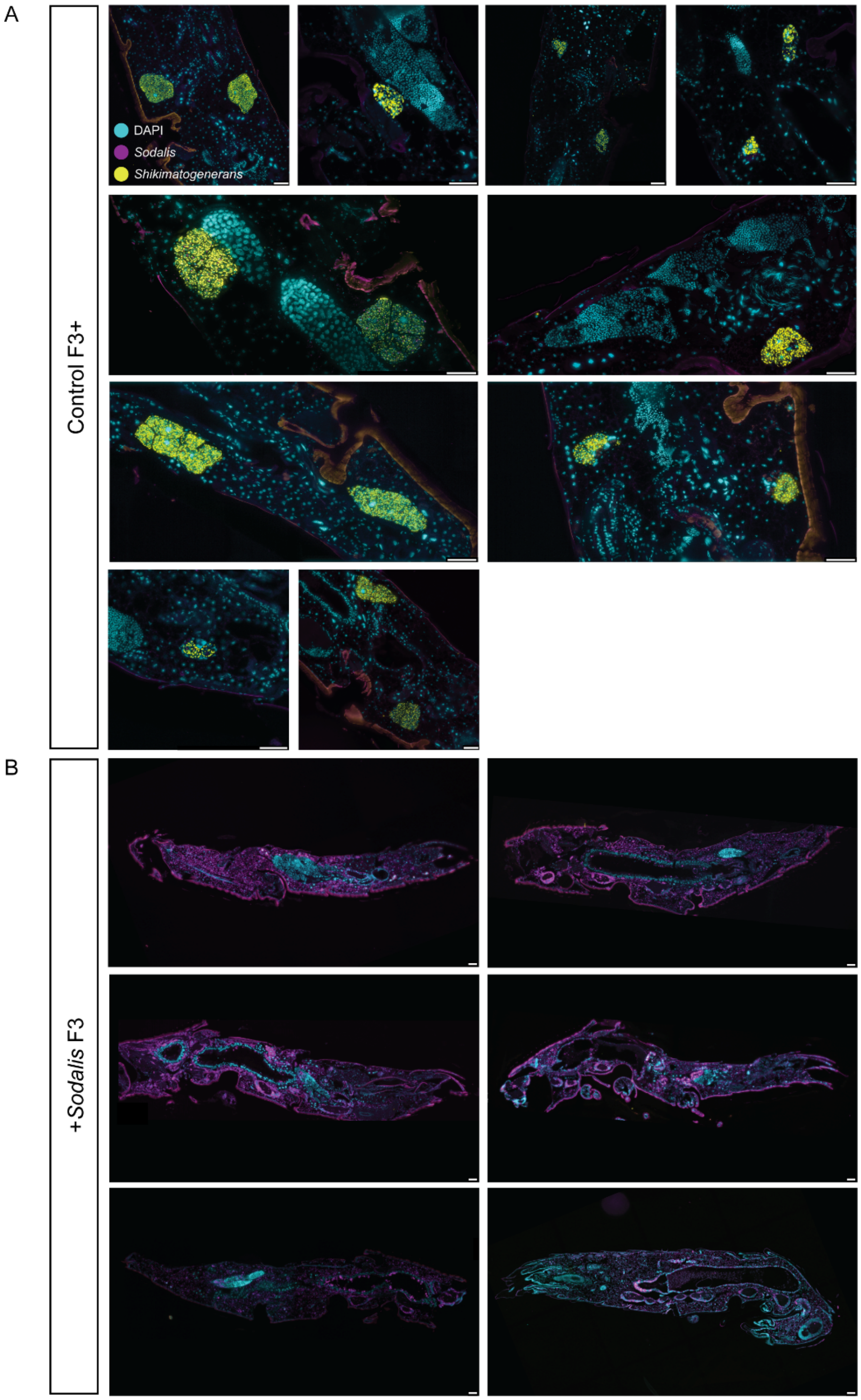

Supplementary Figure 8: FISH micrographs of third generation offspring of control and *Sodalis*-injected female beetles. a Micrographs of third generation offspring of control beetles injected with 0.85% NaCl (w/v) solution (n=10). **b** Micrographs of third generation offspring of beetles injected with *Sodalis*; no *Shikimatogenerans* could be detected in any of the beetles (n=6). DNA was stained with DAPI (cyan), while the probes OsurSym16S-Cy5 and Sod-FISH-Cy3 were used to label *Shikimatogenerans* (yellow) and *Sodalis* (magenta), respectively. Scale bars equal 50 µm.

A

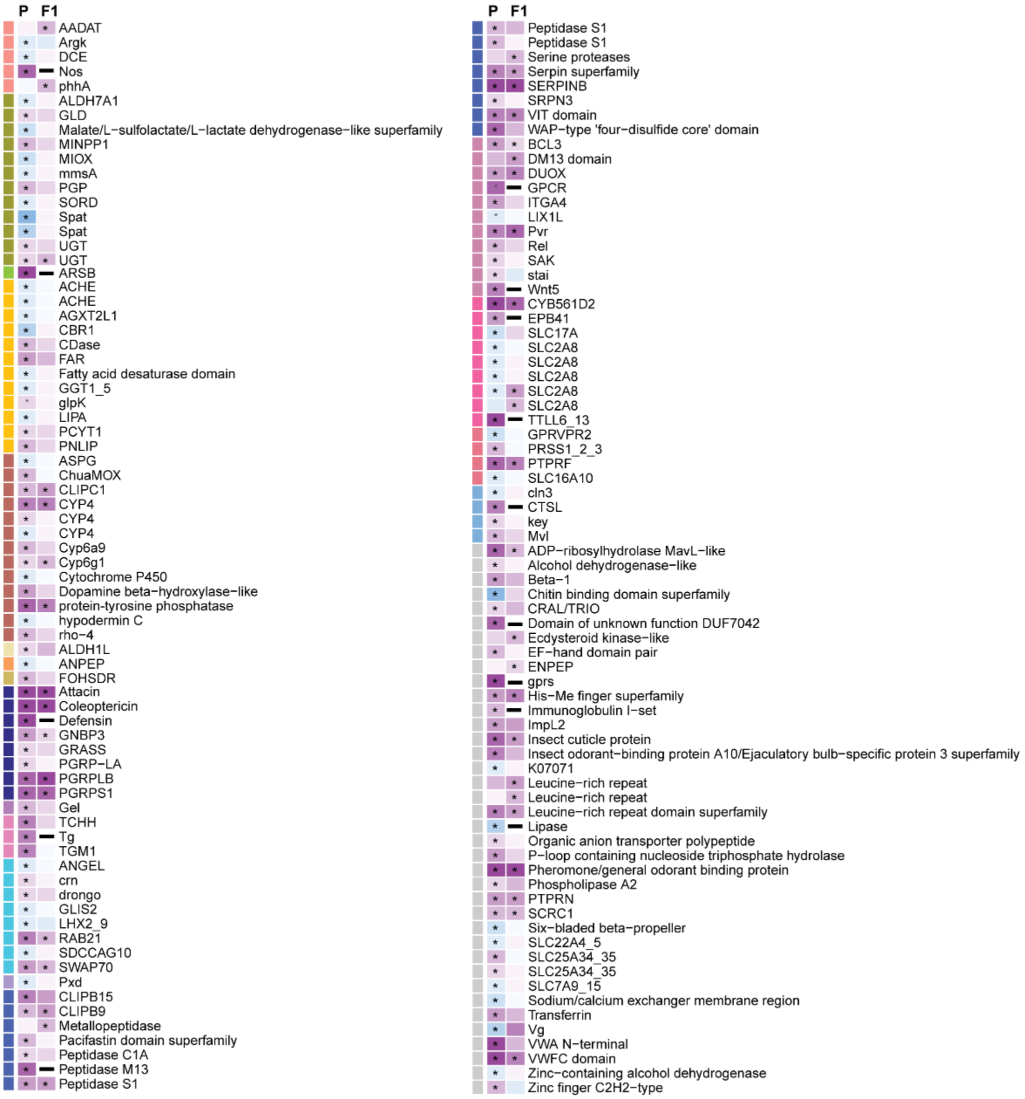

B

	PGRP-LB	
	Control	+*Sodalis*
P	15.47	378.70
F1	2.48	419.33

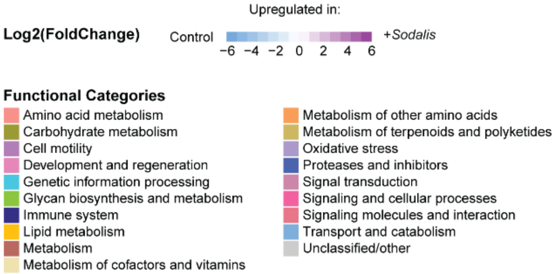

Supplementary Figure 9: Host genes differentially expressed in the bacteriomes upon *Sodalis* infection. a Heat maps of differential gene expression analysis results for control vs. *Sodalis*-infected *O. surinamensis* in the parental (P, n=7) and first offspring (F1, n=7) generations. Asterisks represent significant upregulation of genes in control (cyan) or *Sodalis* infected (purple) beetles (FDR <0.05, log2(fold change) > |1|). Intensity of purple and cyan coloration of boxes indicates log2-fold change in expression of the respective genes. Genes without any informative annotation were not included, genes that were only included in the analysis of only one generation are indicated by a dash. **b** Mean number of reads assigned to host PGRP and PGRP-LB in control vs. *Sodalis*-infected *O. surinamensis* in the parental (P) and first offspring (F1) generation. Source Data are provided as a Source Data file.

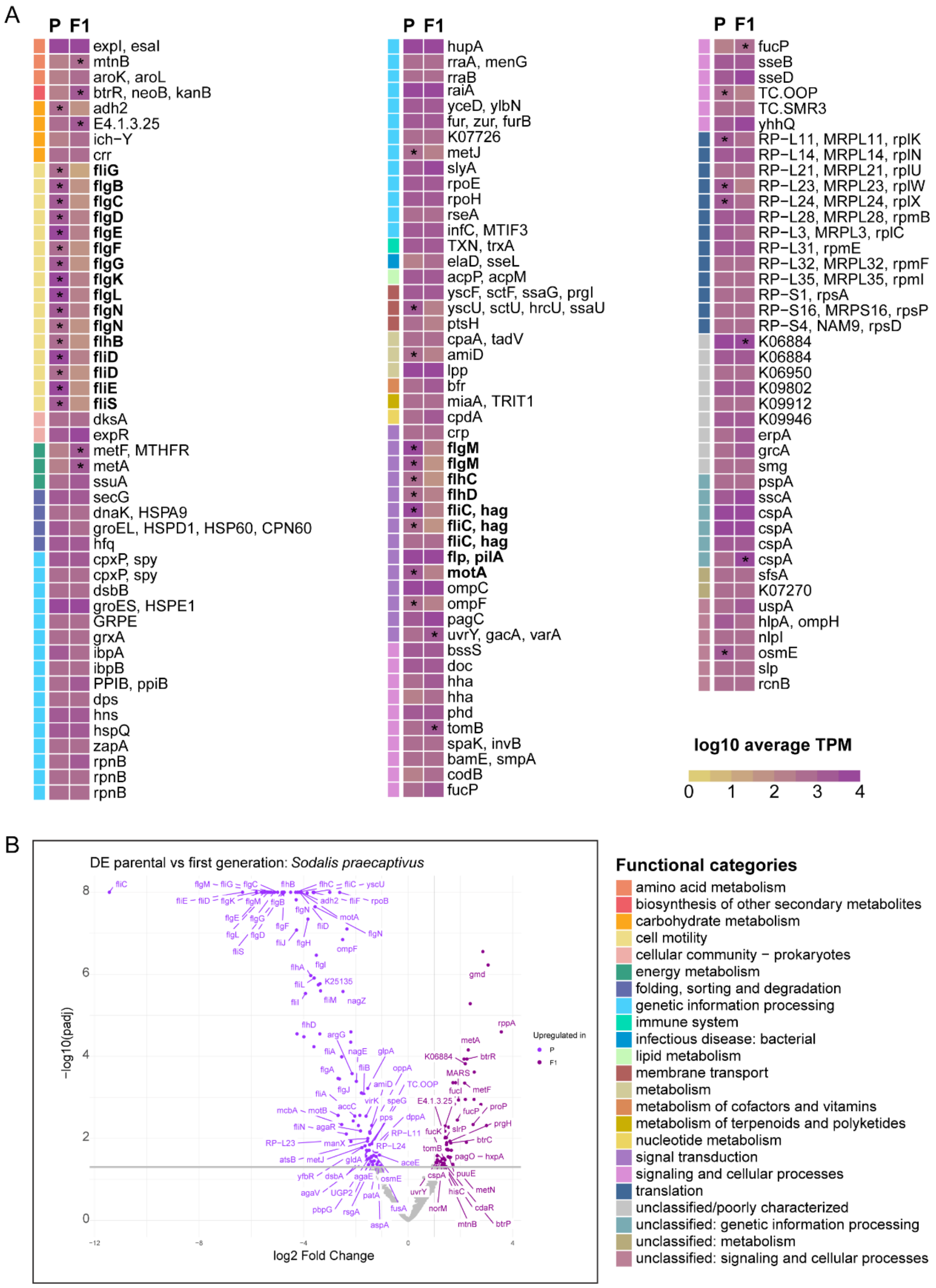

Supplementary Figure 10: Genes expressed by *Sodalis* in the host bacteriomes. a Heat map displaying the combined 100 most highly expressed genes of *Sodalis* in bacteriomes of *O. surinamensis* beetles of the parental (n=7) and the first offspring generation (n=7). Color scale from yellow to purple indicates log10 average TPM of the respective genes. Genes involved in motility are highlighted in bold. **b** Volcano plot of differential gene expression analysis results for *Sodalis* in the bacteriomes of *O. surinamensis* beetles. Lines represent the thresholds for gene expression regarding log2fold change (=|1|) and -log10 of adjusted p-value (0.05, Wald test with Benjamini–Hochberg correction for multiple testing). Source Data are provided as a Source Data file.
