# Supplementary material for: Experimental Sodalis infection eliminates ancient insect symbiont
Source: Nat Commun. 2026 Mar 31;17:3153. doi: 10.1038/s41467-026-71143-2 (PMC13043703; doi:10.1038/s41467-026-71143-2)
Supplement: Supplementary file 2 — Reporting Summary [file 41467_2026_71143_MOESM2_ESM.pdf]

nature portfolio

Corresponding author(s): Kaltenpoth, Martin

Last updated by author(s): Feb 16, 2026

Reporting Summary

Nature Portfolio wishes to improve the reproducibility of the work that we publish. This form provides structure for consistency and transparency in reporting. For further information on Nature Portfolio policies, see our Editorial Policies and the Editorial Policy Checklist.

Statistics

For all statistical analyses, confirm that the following items are present in the figure legend, table legend, main text, or Methods section.

n/a	Confirmed	
☐	☒	The exact sample size (*n*) for each experimental group/condition, given as a discrete number and unit of measurement
☐	☒	A statement on whether measurements were taken from distinct samples or whether the same sample was measured repeatedly
☐	☒	The statistical test(s) used AND whether they are one- or two-sided<br>*Only common tests should be described solely by name; describe more complex techniques in the Methods section.*
☐	☒	A description of all covariates tested
☐	☒	A description of any assumptions or corrections, such as tests of normality and adjustment for multiple comparisons
☐	☒	A full description of the statistical parameters including central tendency (e.g. means) or other basic estimates (e.g. regression coefficient) AND variation (e.g. standard deviation) or associated estimates of uncertainty (e.g. confidence intervals)
☐	☒	For null hypothesis testing, the test statistic (e.g. *F*, *t*, *r*) with confidence intervals, effect sizes, degrees of freedom and *P* value noted<br>*Give P values as exact values whenever suitable.*
☒	☐	For Bayesian analysis, information on the choice of priors and Markov chain Monte Carlo settings
☒	☐	For hierarchical and complex designs, identification of the appropriate level for tests and full reporting of outcomes
☒	☐	Estimates of effect sizes (e.g. Cohen's *d*, Pearson's *r*), indicating how they were calculated

Our web collection on statistics for biologists contains articles on many of the points above.

Software and code

Policy information about availability of computer code

Data collection	- qPCR programme: CFX Manager v3.1 (Bio-Rad, USA)<br>- Micro-computed tomography: Skyscan1272 Control Program v1.5 (Bruker, USA)
Data analysis	- Analysis of cuticle melanization: Natsumushi software v1.10 (https://staff.aist.go.jp/t-fukatsu/Natsumushi.html)<br><br>- Micro-computed tomography:<br>(1) Skyscan1272 Control Program v1.5 (Bruker, USA)<br>(2) NRecon v2.1.0.1 (Bruker, USA)<br>(3) Dragonfly v2022.2 (https://www.theobjects.com/dragonfly)<br><br>- Microscopy: Leica Application Suite X v3.8.2.27713<br><br>- Genome assembly, annotation and RNASeq analysis:<br>(1) Trim Galore v0.6.10 (https://github.com/FelixKrueger/TrimGalore)<br>(2) Sortmerna v4.3.6 (https://github.com/sortmerna/sortmerna)<br>(3) NF-CORE/RNASeq pipeline v3.18 (https://nf-co.re/rnaseq/3.18.0/)<br>(4) Bakta v1.9.3 (https://github.com/oschwengers/bakta)<br>(5) KEGGREST v1.40.1 (https://bioconductor.org/packages/KEGGREST)<br>(6) R v4.3.3 (https://www.r-project.org/)

April 2023

(7) Bowtie2 v2.4.1 (https://github.com/BenLangmead/bowtie2)
(8) Samtools v1.13 and v1.17 (https://github.com/samtools/samtools)
(9) FADU v1.8.3 (https://github.com/IGS/FADU)
(10) GUPPY v4.0.11 (https://nanoporetech.com/document/Guppy-protocol#guppy-software-overview)
(11) Flye v2.9.2 (https://github.com/mikolmogorov/Flye)
(12) Racon v1.4.13 (https://github.com/lbcb-sci/racon)
(13) Medaka v1.0.3 (https://github.com/nanoporetech/medaka)
(14) ntEdit v1.3.2 (https://github.com/bcgsc/ntEdit)
(15) HISAT v2.2.1 (https://daehwankimlab.github.io/hisat2/download/)
(16) BRAKER v3.0.7 (https://github.com/Gaius-Augustus/BRAKER)
(17) AGAT v1.4 (https://github.com/NBISweden/AGAT)
(18) RepeatMasker v4.1.5 (https://github.com/Dfam-consortium/RepeatMasker)
(19) RepeatModeler v2.0.4 (https://github.com/Dfam-consortium/RepeatModeler)
(20) GFFREAD v0.12.8 (https://github.com/gpertea/gffread)
(21) eggNOG-Mapper v3.4.1 and InterProScan within Omics box v3.4.1
(22) edgeR v4.2.2
(23) DESeq2 v1.40.2 (https://bioconductor.org/packages/release/bioc/html/DESeq2.html)
(24) apeglm v1.22.1 (https://bioconductor.org/packages/release/bioc/html/apeglm.html)
(25) BlastKoala v3 (https://www.kegg.jp/blastkoala/)
(26) BlobTools v1.0 (https://doi.org/10.12688/f1000research.12232.1)
(27) BUSCO v5.2.1 (https://doi.org/10.1093/molbev/msab199)

- Statistics:
(1) R: coxme package v2.2.20 CRAN
(2) R: car package v3.1.2 CRAN
(3) R: pscl package v1.5.9 CRAN
(4) R: stats package v4.2.3 CRAN
(5) R: ggplot2 v3.5.1 CRAN

For manuscripts utilizing custom algorithms or software that are central to the research but not yet described in published literature, software must be made available to editors and reviewers. We strongly encourage code deposition in a community repository (e.g. GitHub). See the Nature Portfolio guidelines for submitting code & software for further information.

Data

Policy information about availability of data

All manuscripts must include a data availability statement. This statement should provide the following information, where applicable:

- Accession codes, unique identifiers, or web links for publicly available datasets
- A description of any restrictions on data availability
- For clinical datasets or third party data, please ensure that the statement adheres to our policy

The annotated beetle and symbiont genomes and RNAseq data are available in the Edmond open data repository of the Max Planck Society under https://doi.org/10.17617/3.MUV1MF. RNAseq data generated in this study has also been deposited In the NCBI Sequence Read Archive under accession number PRJNA1423255 [http://www.ncbi.nlm.nih.gov/bioproject/1423255]. The bacterial genomes used in this study were obtained from NCBI using accession numbers GCF_018200315.1 [https://www.ncbi.nlm.nih.gov/datasets/genome/GCF_018200315.1/] (Shikimatogenerans silvanidophilus) and GCA_000517425.1 [https://www.ncbi.nlm.nih.gov/datasets/genome/GCF_000517425.1/] (Sodalis praecaptivus). Single-molecule long reads for assembling the Oryzaephilus surinamensis genome were obtained from the NCBI Sequence Read Archive under accession numbers SRR12881567 [https://www.ncbi.nlm.nih.gov/sra/SRR12881567]-SRR12881568 [https://www.ncbi.nlm.nih.gov/sra/SRR12881568]. RNAseq data used for annotation of the Oryzaephilus surinamensis genome is available in the NCBI Sequence Read Archive under accession number PRJNA1423469 [http://www.ncbi.nlm.nih.gov/bioproject/1423469].

Research involving human participants, their data, or biological material

Policy information about studies with human participants or human data. See also policy information about sex, gender (identity/presentation), and sexual orientation and race, ethnicity and racism.

Reporting on sex and gender	NA
Reporting on race, ethnicity, or other socially relevant groupings	NA
Population characteristics	NA
Recruitment	NA
Ethics oversight	NA

Note that full information on the approval of the study protocol must also be provided in the manuscript.

Field-specific reporting

Please select the one below that is the best fit for your research. If you are not sure, read the appropriate sections before making your selection.

- [x] Life sciences
- [ ] Behavioural & social sciences
- [ ] Ecological, evolutionary & environmental sciences

For a reference copy of the document with all sections, see nature.com/documents/nr-reporting-summary-flat.pdf

Life sciences study design

All studies must disclose on these points even when the disclosure is negative.

Sample size	We did not use any statistical methods to predetermine sample size. Rather, sample sizes were chosen based on other publications,
Data exclusions	We excluded data from beetles of the first offspring generation that were produced by mothers infected with Sodalis praecaptivus but did not harbor the novel symbiont themselves. Additionally, for our analysis of cuticle melanization, we excluded beetle samples (n=6) from Sodalis praecaptivus infected F1 individuals, where pictures had not been taken under standardized light conditions. Apart from these instances, no data was excluded from our analyses.
Replication	Our experiments were performed using individuals from six independent cohorts. From each cohort, we sampled multiple individuals (biological replicates) for all analyses, except fluorescence-in-situ-hybridization, where for some cohorts, we could only collect one individual. Our results were consistent across the different cohorts and all attempts at replication successful.
Randomization	Allocation of beetles to the experimental groups was random.
Blinding	Investigators were not blinded to beetle treatment during the allocation of beetles to the two treatment groups, survival monitoring and sample collection. We abstained from blinding investigators, since beetles and their offspring had to be checked for infection (eventually revealing their treatment group). Once samples were collected, they received an ID number that did not contain information about the treatment group to not bias further analyses. For any analyses involving microscopy and thus specific labelling of bacteria, treatment identity was still apparent due to the presence/absence of Sodalis praecaptivus.

Reporting for specific materials, systems and methods

We require information from authors about some types of materials, experimental systems and methods used in many studies. Here, indicate whether each material, system or method listed is relevant to your study. If you are not sure if a list item applies to your research, read the appropriate section before selecting a response.

Materials & experimental systems

n/a	Involved in the study	
x		Antibodies
x		Eukaryotic cell lines
x		Palaeontology and archaeology
	x	Animals and other organisms
x		Clinical data
x		Dual use research of concern
x		Plants

Methods

n/a	Involved in the study	
x		ChIP-seq
x		Flow cytometry
x		MRI-based neuroimaging

Animals and other research organisms

Policy information about studies involving animals; ARRIVE guidelines recommended for reporting animal research, and Sex and Gender in Research

Laboratory animals	Oryzaephilus surinamensis beetles were acquired from the Julius-Kühn-Institute/Federal Research Centre for Cultivated Plants (Berlin, Germany) in 2014. Since then, these beetles have been reared on organic oat flakes under controlled conditions of 27-28°C and 60% relative humidity (rH) following a day/night cycle of 16/8 h, respectively. Beetles of the parental generation were of undefined age but were considered to be at least two weeks old, since their cuticles were fully melanized. Beetles of the first offspring generation were samples seven days after eclosion from the pupa. Offspring of the third (+ Sodalis treatment) and following generations (control treatment) were of undefined age since they were not kept individually and we had no means of collecting information about their age.
Wild animals	This study did not involve wild animals.
Reporting on sex	Only female O. surinamensis beetles were experimentally infected with Sodalis praecaptivus in the parental beetle generation. From the first offspring generation on, we included all offspring - independent of sex - in our analyses. Male and female beetles were differentiated morphologically based on Halstead, 2009 (doi:10.1017/S0007485300048665). Male beetles were identified based on the presence of a spike on the hindleg femurs, while females lack this spike. Sex was recorded for respective samples during analyses,

	but we did not analyze our data in a sex-based manner since symbiont titer (and thus cuticle melanization/thickness) is not affected by sex.
Field-collected samples	We did not collect samples in the field.
Ethics oversight	All experiments involving animals were performed according to national and institutional regulations.

Note that full information on the approval of the study protocol must also be provided in the manuscript.

Plants

Seed stocks	-
Novel plant genotypes	-
Authentication	-
